# Elucidation of molecular links between obesity and cancer through microRNA regulation

**DOI:** 10.1186/s12920-020-00797-8

**Published:** 2020-10-30

**Authors:** Haluk Dogan, Jiang Shu, Zeynep Hakguder, Zheng Xu, Juan Cui

**Affiliations:** 1 Systems Biology and Biomedical Informatics Lab, Department of Computer Science and Engineering University of Nebraska-Lincoln, Lincoln, 68588-0115 NE USA; 2 Department of Mathematics and Statistics, Wright State University, Dayton, 45435 OH USA

**Keywords:** MicroRNA, Obesity, Cancer, MicroRNA regulation

## Abstract

**Background:**

Obesity contributes to high cancer risk in humans and the mechanistic links between these two pathologies are not yet understood. Recent emerging evidence has associated obesity and cancer with metabolic abnormalities and inflammation where microRNA regulation has a strong implication.

**Methods:**

In this study, we have developed an integrated framework to unravel obesity-cancer linkage from a microRNA regulation perspective. Different from traditional means of identifying static microRNA targets based on sequence and structure properties, our approach focused on the discovery of context-dependent microRNA-mRNA interactions that are potentially associated with disease progression via large-scale genomic analysis. Specifically, a meta-regression analysis and the integration of multi-omics information from obesity and cancers were presented to investigate the microRNA regulation in a dynamic and systematic manner.

**Results:**

Our analysis has identified a total number of 2,143 unique microRNA-gene interactions in obesity and seven types of cancer. Common interactions in obesity and obesity-associated cancers are found to regulate genes in key metabolic processes such as fatty acid and arachidonic acid metabolism and various signaling pathways related to cell growth and inflammation. Additionally, modulated co-regulations among microRNAs targeting the same functional processes were reflected through the analysis.

**Conclusion:**

We demonstrated the statistical modeling of microRNA-mediated gene regulation can facilitate the association study between obesity and cancer. The entire framework provides a powerful tool to understand multifaceted gene regulation in complex human diseases that can be generalized in other biomedical applications.

**Supplementary Information:**

The online version contains supplementary material available at (10.1186/s12920-020-00797-8).

## Background

Obesity is becoming one of the leading public health crises globally and has posed serious threats to human health and life quality. According to the World Health Organization (WHO), by 2030, more than 1.2 billion people globally will suffer from obesity [1]. In the United States, 35.6 percent of adults are affected by obesity. Obesity is associated with an increased risk of several common human cancers and contributes to between 14 to 20 percent of cancer-related mortality cases, making it the leading preventable cause of cancer [2]. The underlying molecular mechanism of obesity development and relapse as well as the enhancing effects of obesity on cancer potency and progression remain largely unknown. In order to gain more insights on aforementioned unascertained mechanisms, there have been strengthened efforts to study the genetic basis of obesity, as well as the regulatory and epigenetic effects on signaling process.

A group of small non-coding RNAs, called microRNA (miRNAs), are important gene silencers in animals and plants [3–5]. For example, over 2,000 human miRNAs can bind to over 60% endogenous genes [5] in humans and fine tune numerous biological processes related to cell growth and signaling [6]. Increasing evidence has shown strong implication of miRNA in complex human diseases including obesity (e.g., elevated abundance of miR-34 in obese mice reduces NAD+ levels and results in obesity-mimetic outcomes [7, 8]; miR-27, miR-519d, and miR-30c are obesity related [9–11]); metabolic disorder (e.g., miR-33a/b, miR-107, miR-103, and miR-34a regulate different metabolism [12]); pro-inflammatory events (e.g., miR-27b, miR-21, miR-34a, miR-106b, miR-130, miR-15b, miR-155, and miR-200c); aging (e.g., downregulation of miR-24, and miR-221 in the peripheral blood mononuclear cells (PBMCs) of older individuals, and increased expression other 21 miRNAs [13]), and cancers (e.g., miR-200 and miR-205 regulates epithelial to mesenchymal transition [14], miR-21 regulates apoptosis in lung cancer [15] and drives melanoma [16]). It should be noted that such linkages were determined largely based on the alterations of miRNA expression in those diseases or the effect of miRNA manipulation in loss-of-function and gain-of-function studies; only a handful were validated by elaborate target function in vivo.

More recently, studies began to unravel miRNA’s role in the obesity-cancer link. For example, it has been demonstrated that the epigenetic silencing of miR-200c is capable of targeting STAT3-G9a signaling and limiting the malignancy of obesity-related breast cancer [17]. miR-10b was found down regulated in the breast tumors of the obese subjects compared to the lean; the suppression of miR-10b in breast cancer cell line increases cell proliferation and invasion and affects inversely the expression of targets BCL2L11, PIEZO1 and NCOR2 cell [18]. As reported in Kasiappan et al., [19] over 50 miRNAs show altered expressions in either obesity or breast cancer, and miR-302b and miR-498 are differentially expressed in obesity-associated breast cancer. Other examples include a list of miRNAs potentially linking obesity and colorectal cancer, namely miR-425, -196, -155, -150, -351, -16, -34, -148, -4443, -101, -27b, and let-7 [20]. Note that most such discoveries were made based on the expression analysis and the causality of the disease development is not fully validated. Many of the dysregulated miRNAs common in obesity and cancers were evidenced to regulate conditions such as insulin resistance, low-grade inflammation, cell proliferation and survival, dysfunction of adipose tissues, and increased oxidative stress through targeting VEGF, Ras, HIF1- *α*, PI3K/Akt, JAK/STAT3, MAPK, ERK/p38, and NF-k *β* [19, 21–25]. All those events are important elements in obesity associated cancer development. In addition to endogenous regulation, there are growing interest in studying extracellular miRNAs associated with vesicles or protein complexes, e.g., adipose-derived circulating miRNAs, as important regulators in other tissues and diseases [26]. However, a grand challenge here is the lack of systems approaches and reliable high-throughput technologies that can assess miRNA roles in human disease progression in a holistic and dynamic fashion. In this regard, the development of sequencing technologies such as cross-linking, ligation, and sequencing of hybrids (CLASH) [27] and individual-nucleotide resolution CLIP (iCLIP) [28] allows the identification of the exact binding site of the miRNA on mRNAs, however, the drawbacks of the selection bias in base pairing, low coverage, and ambiguous downregulation still hinder the broad application in this research field.

It is known that miRNAs bind to the 3’-UTR of target genes to either trigger mRNA degradation or inhibit protein translation [29, 30]. The interactions between miRNA and genes exhibits a high level of complexity since most miRNAs can bind to multiple gene targets while the genes can be targeted by different miRNAs [31–34]. Additionally, the binding process has been found to be condition-specific, which means that the same miRNA may bind to different groups of mRNAs under different biological contexts. The underlying binding mechanism involves both competition (when the binding sites of different miRNAs are in close or overlapping regions on the target genes) and cooperation (when several miRNAs bind to different regions of the same target) [31–34]. This type of dynamic feature is hardly captured by early computational tools such as PicTar [32], MicroTar [35], miTarget [36], TargetRNA2 [37], TargetScan [38], and miRwalk [39], which utilize mainly sequence and structure characteristics to build the models. The second-generation target prediction tools integrate gene and/or miRNA expression profiles into the modeling process to reflect condition-specific mechanisms, including MiRonTop [40], mirAct [41], CoSMic [42]. Negatively correlated expressions between miRNA and their target genes are largely used as a strong indicator for real interaction in those methods. Considering the complicated machinery in gene expression regulation that involves transcription factor (TFs), methylation, and genetic variations, current research is exploring systems approaches that can take into consideration of these mechanisms underlying the multifaceted gene regulation and competitive miRNA binding.

In this article, we conducted a meta-analysis of miRNA regulation on both obesity and cancer to unravel the molecular association between these two types of disease. In order to investigate context-dependent miRNA-gene interactions, a new meta-regression method was presented to integrate large-scale genomics profiles in respective diseases, such as gene and miRNA expression, copy number variation (CNV), and DNA methylation. The inferred interactions were compared with literature and other resources. For example, we further examined liver cancer interactions through analyzing our in-house gene expression data in a liver disease mouse model. We present the association between obesity and different types of cancers through identifying common condition-associated miRNA-mRNA interactions and characterizing regulatory transition across diseases.

## Methods

**Fig. 1:**
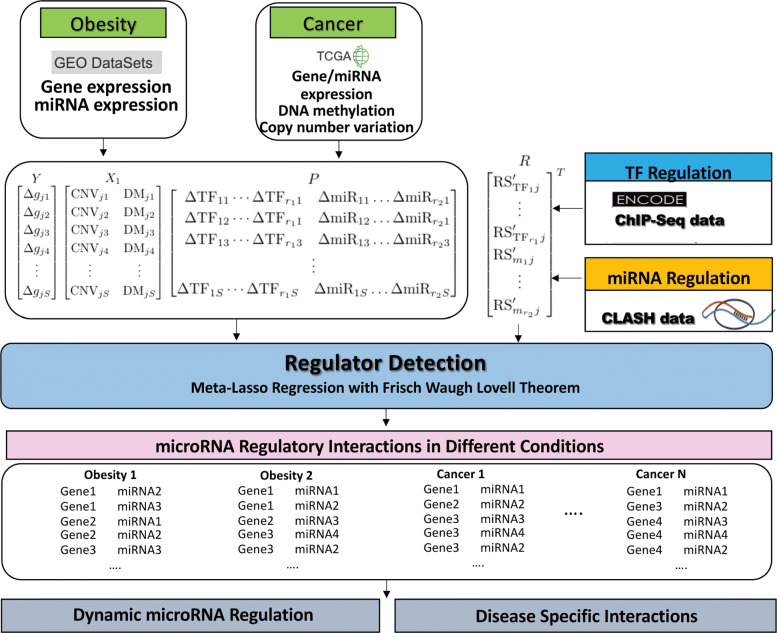
The overview of the analytic workflow

In this section, a detailed description of the proposed computational framework is provided, including data collection and processing, binding potential estimation, and the regression-based model for miRNA-gene interaction identification. The schematic analytical workflow is shown in Fig. 1.

### Genomic expression profiles

In this study, the genomic data were collected from the following resources:

1. GSE53378 and GSE25402 on a total of 42 obese patients from the Gene Expression Omnibus (GEO);
2. A collection of 3,484 patients with 7 major types of cancer (1,279 early stage patients) from The Cancer Genome Atlas (TCGA).

Names and download links of datasets used in our experiments are listed in Table 1. All cancer types were categorized into strong and weak association groups, SAG and WAG, respectively, according to their hazard ratios of the association with obesity [43]. Table 2 contains the detailed statistics of the obesity and cancer samples used in this study. For each sample, the expression profiles of gene and miRNA were extracted while information about copy number variation and DNA methylation was also collected for each cancer sample. Note that the dataset includes both microarray and sequencing data from different disease conditions. In order to make the results meaningfully comparable, we make sure each data has its own control (either paired or unpaired with diseased samples), as well as matched miRNA and mRNA expression profiles from the same technology (either microarray or sequencing). Within each disease, normalized expression was used to calculate the expression change required for the downstream analysis.

**Table 1:** Names and download links of datasets used in our experiments

Dataset name	Download link
GSE53378	https://go.unl.edu/microarray-pre
GSE25402	https://go.unl.edu/microarray-obese
TCGA-BRCA	https://go.unl.edu/tcga-brca
TCGA-KIRC	https://go.unl.edu/tcga-kirc
TCGA-KIRP	https://go.unl.edu/tcga-kirp
TCGA-LIHC	https://go.unl.edu/tcga-lihc
TCGA-LUAD	https://go.unl.edu/tcga-luad
TCGA-LUSC	https://go.unl.edu/tcga-lusc
TCGA-UCEC	https://go.unl.edu/tcga-ucec
hg19	https://go.unl.edu/hg19
miRNA-mRNA interactions	https://go.unl.edu/clash

**Table 2:** The detailed statistics of obesity and cancer datasets

	**Obesity**		Normal	Obese
	Adipose tissue (pre- and post-surgery)	OB1	16	16
	Adipose tissue (obese and lean)	OB2	30	26
	**Cancer**		**Normal**	**Cancer (Early Stage)**
**S A G**	Kidney renal clear cell carcinoma	KIRC	72	533 (251)
	Kidney renal papillary cell carcinoma	KIRP	32	290 (140)
	Liver hepatocellular carcinoma	LIHC	50	371 (164)
	Uterine corpus endometrial carcinoma	UCEC	24	176 (97)
**W A G**	Breast invasive carcinoma	BRCA	114	1097 (124)
	Lung adenocarcinoma	LUAD	59	515 (276)
	Lung squamous cell carcinoma	LUSC	51	502 (227)

### Interactome profiles and data preprocessing

We downloaded the raw sequencing data of experimental miRNA-mRNA interactions from the CLASH analysis [27], as well as ChIP-Seq data on 150 Transcription factor (TFs) from the Encyclopedia of DNA Elements (ENCODE) Consortium [44].

First, the raw data from CLASH was re-mapped against human reference genome (hg19). Three types of reads were examined: 1) chimeric reads that contain miRNA-miRNA binding sites; 2) miRNA single reads that represent unbound miRNAs; 3) mRNA single reads that represent unbound mRNAs. After the read counts of each type were calculated, we utilized the binomial test to infer the confidence level of an identified binding site *k* between miRNA *i* and mRNA *j* with respect to a random observation. A *p*-value was calculated as follows:

$$p\text{-value}_{ijk}=\sum_{c={ C }_{ijk} }^{{ C }_{\text{all}} } \left(\begin{array}{cc} {{C}_{\text{all}}}\\ {c} \end{array}\right){ \left(\frac { { C }_{ijk} }{ { C }_{\text{all}} } \right)^{c }}{ \left(1-\frac { { C }_{ijk} }{ { C }_{\text{all}} } \right)^{{ C }_{\text{all} }-c}} $$

where *C*_*all*_ denotes the total of supporting chimeric reads of all interactions; *C*_*ijk*_ is the count of supporting chimeric reads of interactions between miRNA *i* and mRNA *j* at the binding site *k*.

Binding sites with Bonferroni-multiple-test-adjusted *p*-value less than 0.05 were considered to be significant and included in the further analysis. For quality control, two additional filters were applied: 1) eliminating the bottom 10% of low confidence binding sites; 2) discarding the binding sites if the number of supporting chimeric reads is less than 12.

### Estimation of regulatory score (RS)

As described above, we suspected that different regulators shared an equal possibility to interact with a common target. Therefore, we designed a procedure to qualitatively estimate the regulatory potentials between regulators and their targets.

#### RS of miRNA-gene interactions

First, we calculated the probability of a binding event between miRNA *i* and gene *j* at the binding site *k* through a conditional probability:

$$ P_{ijk}=\frac{{C_{i j k}}^{2}}{\left(C_{i} + M_{i}\right)\left(C_{j} + M_{j}\right)} $$

where *C*_*i*_ and *M*_*i*_ are the total of chimeric reads and single reads associated with miRNA *i*, respectively; similarly, *C*_*j*_ and *M*_*j*_ indicate the total of interactions and free mRNA related to mRNA *j*, respectively.

Then, the RS of a miRNA-gene pair was calculated based on the aggregation of the binding probability and the binding affinity, e.g., represented by the minimum free energy (MFE) [45] of the interaction, as follows:

$$\text{RS}_{ij}=\frac{1}{K}\sum_{k=1}^{K}\left|\text{MFE}_{k}\right| \times P_{ijk}) $$

where MFE_*k*_ represents the minimum free energy of the binding site *k* and *K* is the total number of different binding sites between miRNA *i* and gene *j*.

Considering the competition among all possible targets of a miRNA regulator, RS was further normalized by the total number of interactions associated with miRNA *i*:

$$\text{RS}_{ij}^{\prime}=\frac{\text{RS}_{ij}}{\text{RS}_{i}} $$

where RS_*i*_ denotes the sum of RS between miRNA *i* and all its target.

#### RS of TF-gene interactions

Unlike the RS of a miRNA-gene pair was calculated based on abundances, the RS for the TF *t* and gene *j* was estimated based on the distance, *d*, between the TF binding site *k* and the transcription start site (TSS) [46, 47], as follows:

$$\text{RS}_{tjk}=e^{-\left(0.5+4\left(\frac{d}{10^{5}}\right)\right)} $$

Then, the RSs for all binding sites between a TF and gene pair (*t* and *j*) can be aggregated by [46]:

$$\text{RS}_{tj}=1-\prod_{k=1}^{K}\left(1-\text{RS}_{tjk}\right) $$

Similarly, considering that the same TF can bind to several different genes, RS was normalized as $\text {RS}_{tj}^{\prime }=\frac {\text {RS}_{tj}}{\text {RS}_{t}}$ where RS_*t*_ denotes the sum of RS between TF *t* and all regulated genes.

### The meta-regression model for gene regulation identification

In this study, each disease was considered as a specific condition $D_{t}\ (t=1, 2,\dots, 9)$, which includes two conditions for obesity and seven cancers. For each condition *D*_*t*_, we created four matrices for each gene as shown in Equations 1 and 2.

1 $$\begin{array}{*{20}l} \renewcommand\arraystretch{1.5} &\stackrel{Y}{ \left[\begin{array}{cccccc} \Delta { { g }_{j1 }} \\ \Delta { { g }_{j2 }} \\ \Delta { { g }_{j3 }} \\ \Delta { { g }_{j4 }} \\ \vdots \\ \Delta { { g }_{jS }} \\ \end{array}\right] }\, \stackrel{\text{X}_{1}}{ \left[\begin{array}{cccccc} \text{CNV}_{j1} & \text{DM}_{j1} \\ \text{CNV}_{j2} & \text{DM}_{j2} \\ \text{CNV}_{j3} & \text{DM}_{j3} \\ \text{CNV}_{j4} & \text{DM}_{j4} \\ \vdots & \vdots \\ \text{CNV}_{jS} & \text{DM}_{jS} \\ \end{array}\right] }\, \stackrel{R}{ \left[\begin{array}{cccccc} \text{RS}'_{\text{TF}_{1}j} \\ \multicolumn{1}{c}{\vdots} \\ \text{RS}'_{\text{TF}_{r_{1}}j} \\ \text{RS}'_{m_{1}j} \\ \multicolumn{1}{c}{\vdots} \\ \text{RS}'_{m_{r_{2}}j} \\ \end{array}\right]^{T} }  \end{array} $$

2 $$\begin{array}{*{20}l} \renewcommand\arraystretch{1.5} &\stackrel{P}{ \left[\begin{array}{cccccc} P^{1} \\ P^{2} \\ P^{3} \\ \vdots \\ P^{S} \end{array}\right] }\, \stackrel{P}{ \left[\begin{array}{cccccc} \Delta { { \text{TF} }_{1 1} } \cdots \Delta { { \text{TF} }_{r_{1} 1}} \quad \Delta { { \text{miR} }_{1 1} } \dots \Delta { { \text{miR} }_{r_{2} 1}} \\ \Delta { { \text{TF} }_{1 2} } \cdots \Delta { { \text{TF} }_{r_{1} 1} } \quad \Delta { { \text{miR} }_{1 2} } \dots \Delta { { \text{miR} }_{r_{2} 1} } \\ \Delta { { \text{TF} }_{1 3} } \cdots \Delta { { \text{TF} }_{r_{1} 3} } \quad \Delta { { \text{miR} }_{1 3} } \dots \Delta { { \text{miR} }_{r_{2} 3} } \\ \multicolumn{1}{c}{\vdots} \\ \Delta { { \text{TF} }_{1 S} } \cdots \Delta { { \text{TF} }_{r_{1} S}} \quad \Delta { { \text{miR} }_{1 S} } \dots \Delta { { \text{miR} }_{r_{2} S} } \\ \end{array}\right] }  \end{array} $$

#### Expression matrix

*Y*(*S*×1) contains the expression changes of gene *j* in each of the *s* samples associated with *D*_*t*_ compared to the control group. Here, the expression changes of gene *j* in sample *s* were calculated as follows:

$$\renewcommand\arraystretch{1.5} \Delta {{ g }_{js }}=\log_{2 }{\frac { { g }_{js }} {\overline{{ g }_{j \text{ctrl}}}} }$$

#### The background matrix

*X*_1_(*S*×2) includes values of the CNV and methylation change of the gene *j* in each sample versus the normal group. It must be noted that, in this study, the background matrix *X*_1_ is only constructed for cancer conditions as CNV and DNA methylation information were not available for obesity dataset.

#### The regulator matrix

*P*(*S*×*R*) contains the expression changes of all TF and miRNA regulators of gene *j* in *s* samples versus the control group. *R* denotes the total number of regulators of gene *j*.

#### The regulatory score matrix

*R*(1×*R*) contains the RS between gene *j* and its regulators (TFs and miRNAs). *R* denotes the total number of regulators of gene *j*.

We constructed a linear regression model [7] with a updated matrix $X_{2}\ ([P^{1} \times R, \dots, P^{S} \times R]^{T}$, which are the products of R and each row in P) to represent the regulatory effects of TF and miRNA. Since the background matrix *X*_1_ is not our focus, the Frisch–Waugh–Lovell (FWL) method was employed to transform the two-component regression model [7] to a standard linear regression model [8]. Through this linear transformation, the expression changes of gene (Y) were adjusted by $M_{X_{1}}$, which presents the influences of CNV and methylation on gene expression change. Last, the linear regression model [8] was solved with variable selection by using Mallows’s Cp method [48].

$$\begin{aligned} Y &= X_{1}\beta_{1}+X_{2}\beta_{2}+\epsilon\\ M_{X_{1}}Y &= M_{X_{1}}(X_{2}\beta_{2}+\epsilon) \end{aligned} $$

where $M_{X_{1}}=I-X_{1}(X_{1}^{T}X_{1})^{-1}X_{1}^{T}$. The statistical significance of each regulator in the final model was assessed based on the consistency across all samples under the same condition. False discovery rate (Benjamini-Hochberg procedure, Q-value < 0.05) [49] was used to remove any insignificant regulators of corresponding gene.

### Sampling for consensus regulator selection

To minimize the variation on regulator detection, we repeated the sampling process 20 times and generated 20 randomized datasets. Then, for each randomized dataset (*U*_*i*_), we conducted regulator detection through the aforementioned model and obtained regulators of gene *j*(*W*_*ij*_). On the other hand, the original dataset (*U*_org_) was also applied on the regression-based model to obtain a set of regulator of gene *j*,*W*_org-j_. The regulators which were in *W*_org-j_ and have been consistently detected in all *W*_*ij*_, are considered as the consensus regulators of gene *j*. As the final result, *W*_con−*j*_ that contains all consensus regulators of gene *j* is reported. For each reported interaction, a summarized coefficient and consensus measure represented by a percentage were given to reflect the selection consistency and confidence. The rationale is as follows. When regulatory interaction is formulated as a regression problem where target gene is a response variable and regulators are predictor variables, the regression coefficients, *β*, obtained after optimization indicate a relationship. Nonzero *β* values show existence of such relationship. Proportion of correctly estimated nonzero *β* values shows the sensitivity, and that of zero *β* values shows specificity. Without the ground truth of the relationships, calculation of sensitivity and specificity is not possible for method performance evaluation. Therefore, we define a consistency metric to show the confidence in our model predictions. Regression coefficients from Meta-lasso model was obtained for target gene and its regulators in each data split. Consistency is the proportion of nonzero regression coefficients in all the splits. We set a threshold at 0.7 for consistency values.

### Over-sampling of the obesity dataset

Since our model considers all major regulators in this meta-regression analysis, it requires a minimal sample size for the reliable detection of gene regulators, which is challenging for the obesity datasets. To overcome this problem, an over-sampling process was implemented. The basic idea is to generate new samples by introducing random small variations to the existing samples in the original dataset to maintain the same sample distribution. As showed in [9], *E*_*s*_ is the whole genomic profile of one original sample *s* and *V*_*σ*_ is a randomly generated variation vector, which has the same dimension as *E*_*s*_. The elements in variation vector *V*_*σ*_ were randomly sampled from 0.95 to 1.05. Then, the new sample $E^{\prime }_{s}$ was created as follows:

$$ E'_{s} = E_{s} \times V_{\sigma} = [E_{s1}V_{\sigma 1},E_{s2}V_{\sigma 2}, \dots, E_{si}V_{\sigma i},\dots]\, $$

where *V*_*σi*_∼Uniform(0.95,1)

Subsequently, the same analysis was performed for every human gene $g_{j}\ (j=1, 2, \dots, 20531)$ in obesity and seven cancers.

## Results

First, through the aforementioned modeling and data analysis, we identified the differentially expressed (DE) genes and miRNAs (with Fold Change (FC) >=2 and *p*-value < 0.05 through t-test) in each cancer and obesity, as well as the miRNA-gene interactions in each condition. The detailed statistics are summarized in (Supplementary Table 1) while the full list of interactions is at http://sbbi-panda.unl.edu:8001/obesity-regulators/.

### Common DE genes and miRNA regulations reflect associations between obesity and cancers

To investigate the molecular association between obesity and different cancers, we first looked into the DE-genes and -miRNAs in each diseased condition versus the corresponding normal. In general, there are less DE-genes and -miRNAs in obesity than in cancers. For example, 405 genes and 9 miRNAs showed significant expression changes (FC >=2) in obese group compared to normal while in average, there are 8,988 DE genes, and 29 DE miRNAs with FC >= 2 and 1,035 with FC >1 in cancers (5,425 DE-genes and 120 DE-miRNAs identified in the early stage of cancer).

Fewer DE-genes were identified in early stage cancers from SAG (Mean = 5,296, Confidence Interval = [4,242, 6,244]) than WAG (Mean = 5,599, Confidence Interval = [4,711, 6,948]). Surprisingly, even with fewer DE-genes, the SAG still consistently shares more common DE-genes with obesity than the WAG, i.e. [41.2%, 59.3%] versus [27.0%, 38.3%] as shown in Table 3. For example, 59.3% of DE-genes in obesity were also in early stage kidney cancer (KIRC), while only 27% of DE genes are common between obesity and early stage lung cancer (LUSC).

**Table 3:** The Overview of Commonality Between Obesity and Cancers (A, B% denotes the number of overlapped genes/miRNAs/interactions between obese and cancer, and % of obese is overlapped with the corresponding cancer)

		Obesity	Strong association group				Weak association group		
			KIRC	KIRP	LIHC	UCEC	BRCA	LUAD	LUSC
Hazard ratio (HR) [32]		-	1.25		1.19	1.62	0.97	0.82	
Differential expression	Genes	405	240,	205,	168,	167,	130,	155,	109,
			59.3%	50.6%	41.5%	41.2%	32.1%	38.3%	27.0%
	miRNAs	9	2,	3,	1,	3,	2,	2,	2,
			22.2%	33.3%	11.1%	33.3%	22.2%	22.2%	22.2%
MicroRNA regulatory interactions	Interactions	1558	523,	556,	497,	568,	492,	534,	507,
			31.6%	33.6%	31.9%	36.6%	31.6%	34.3%	32.5%
	Genes	1355	806,	845,	776,	809,	770,	846,	848,
			59.5%	62.4%	57.3%	59.7%	56.8%	62.4%	62.6%
	miRNAs	180	110,	115,	115,	112,	111,	108,	107,
			61.1%	63.9%	63.9%	62.2%	61.7%	60.0%	59.4%

It has been known that not many miRNAs change in the adipose cells in obesity. As shown in our analysis, only 9 miRNAs were found differentially expressed in obesity, with FC >=2. There is no evidence suggesting the significant commonality between SAG and obesity in terms of DE-miRNAs. Note that those reported in obesity-cancer linkage shows consistent expression patterns, e.g., let-7 and miR-10b are down regulated in obesity and breast cancer). However, considering the fact that different miRNAs may co-regulate the same gene targets and lead to the same crucial functional changes, it is highly warranted to investigate the obesity-cancer associated in terms of miRNA regulatory interactions.

By examining the miRNA-gene interactions detected in each disease, we found that miRNA regulations are highly involved in both obesity and early stage cancers (consistent across all types of cancers). About 92% (out of 1,558) miRNA-gene interactions in obesity were detected in at least one type of cancer as shown in Table 3. More than half of miRNA-mediated genes in obesity were also targets of miRNAs in cancers. Observations also suggest that the same miRNAs may interact with different gene targets under different disease conditions.

### miRNA regulated pathways in obesity and early-stage SAG cancers

To demonstrate that miRNA regulation is involved in the link between obesity and cancers, particularly at the early stage, we conducted gene set enrichment analysis [50] to identify the altered functional pathways based on miRNA targets in each dataset. As shown in Table 4, we listed the top five enriched pathways from three major functional categories that are critical in obesity and cancers, such as signaling pathways, metabolism pathways and inflammation pathways. We found that the patterns in some particular pathways can differentiate the SAG and WAG. For example, MAPK signaling pathway was consistently enriched in down-regulated genes in three cancers of WAG while being enriched in up-regulated genes in obesity, kidney and liver cancers. Similarly, two pathways related to fatty acid metabolism and glycine, serine and threonine metabolism also showed common alterations among obesity, kidney cancer, and liver cancer. With respect to immune response, obesity showed higher consistency with SAG than WAG.

**Table 4:**
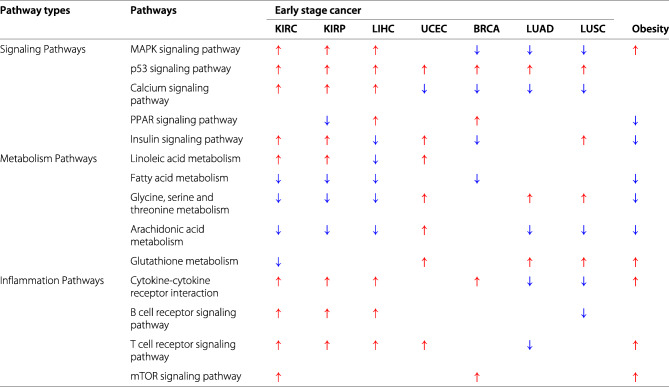
Functional pathways enriched in obesity and early stage cancers in the selected categories ( and  indicate that the pathways were identified among the up-regulated and down-regulated genes, respectively, in the corresponding condition; those in bold show consistent patterns between obesity and SAG cancers)

### miRNAs co-regulate the same biological process by targeting different genes in each disease

**Fig. 2:**
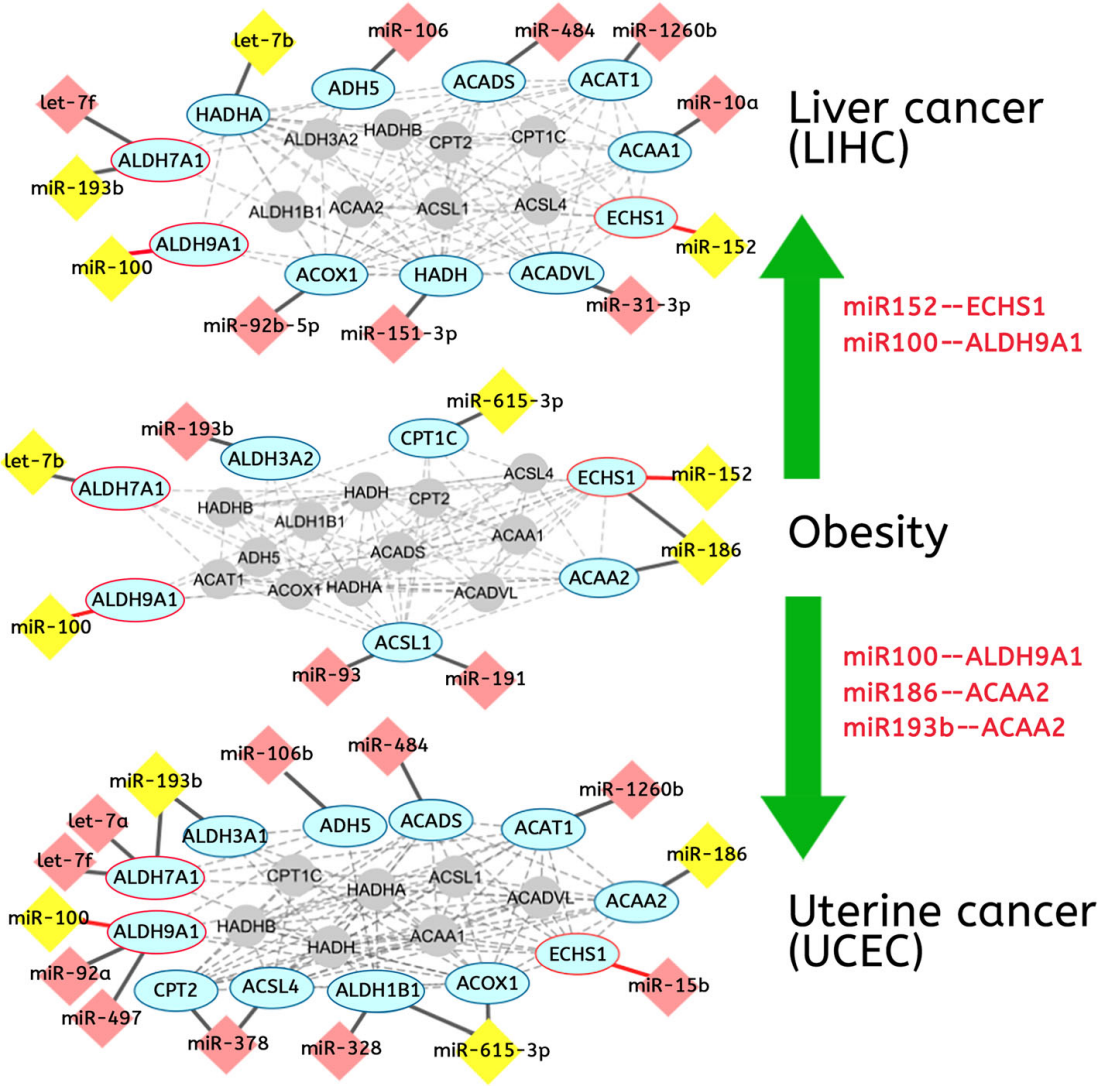
Dynamic regulation of miRNA in fatty acid metabolism in obesity, early-stage liver and uterine cancers

Next, we compared obesity with early-stage SAG cancers and identified 31 common miRNA-gene interactions and 9 common critical pathways, as shown in Table 5. Taking fatty acid metabolism as an example, 8 miRNAs and 7 genes are involved in obesity as shown in the middle panel of Fig. 2. Between obesity and early-stage liver cancer, upper panel of Fig. 2, two interactions (miR-152/ECHS1 and miR-100/ALDH9A1) remained the same (in red) and another two common miRNAs (miR-193b and let-7b) regulate different gene targets in LIHC compared to obesity. Specifically, miR-193b regulates ALDH3A2 and let-7b interacts with ALDH7A1 in obesity, however, in liver cancer, miR-193b regulates ALDH7A1 and let-7b targets HADHA, they regulate different genes (miR-193b /ALDH7A1, let-7b/HADHA) that participate in the same functional process. When looking at the fatty acid metabolism in obesity and uterine cancer, three miRNA-gene interactions (miR100/ALDH9A1, miR-186/ACAA2 and miR- 193b/ALDH3A2) were shared in common. In addition, another 5 common genes and 4 miRNAs were involved with different interactions. For example, miR-615-3p interacts with CPT1C in obesity and regulates ACOX1 and ALDH1B1 in uterine cancer.

**Table 5:** Common miRNA Regulatory Interactions and Enriched Pathways Between Obesity and Cancer of SAG

	Strong association group
Common obesity- detected miRNA regulatory interactions	miR-320a – MLL/OTUD1/PPIAL4G/PPP1R7;
	miR-18a – CSDA/IRS4/KLHDC10;
	miR-193b – GMPR2/MYST3/ZNF71;
	miR-484 – FAM128B/NOMO3/TMEM93;
	miR-615-3p – KIAA0494/LRRC37A2/MRPL23;
	miR-17 – CBL/TRAP1;
	let-7b – SPTBN2; let-7e – EIF2C2;
	miR-106a – HDHD1A; miR-125b – GPRIN1;
	miR-149 – JMJD5; miR-186 – INPP5F;
	miR-192 – KIAA1671; miR-378 – MLF1;
	miR-935 – TALDO1; miR-625 – LASS2;
	miR-30e – EXO1; miR-671-5p – CCDC21;
	miR-1301 – ZCCHC24
Enriched pathways through miRNA regulations	Fatty Acid Metabolism
	Arachidonic Acid Metabolism
	Purine Metabolism
	MAPK Signaling Pathway
	P53 Signaling Pathway
	Axon Guidance
	Focal Adhesion
	Gap Junction
	Leukocyte Transendothelial Migration

**Fig. 3:**
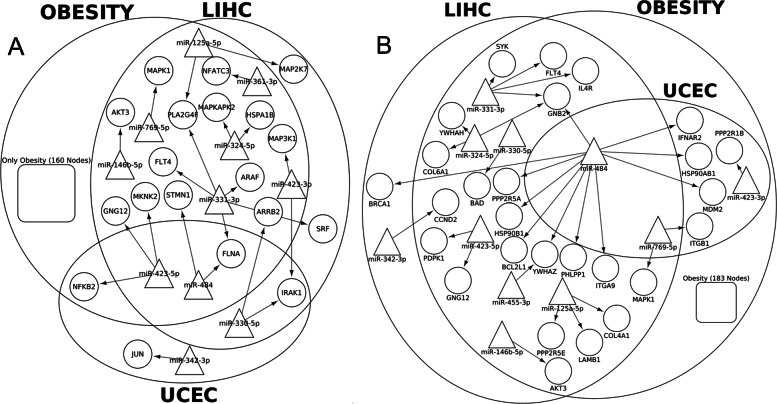
MiRNA regulation in signaling processes in obesity, liver and uterine cancers. **a**) MAPK signaling pathway and **b**) PI3K-Akt signaling pathway

**Fig. 4:**
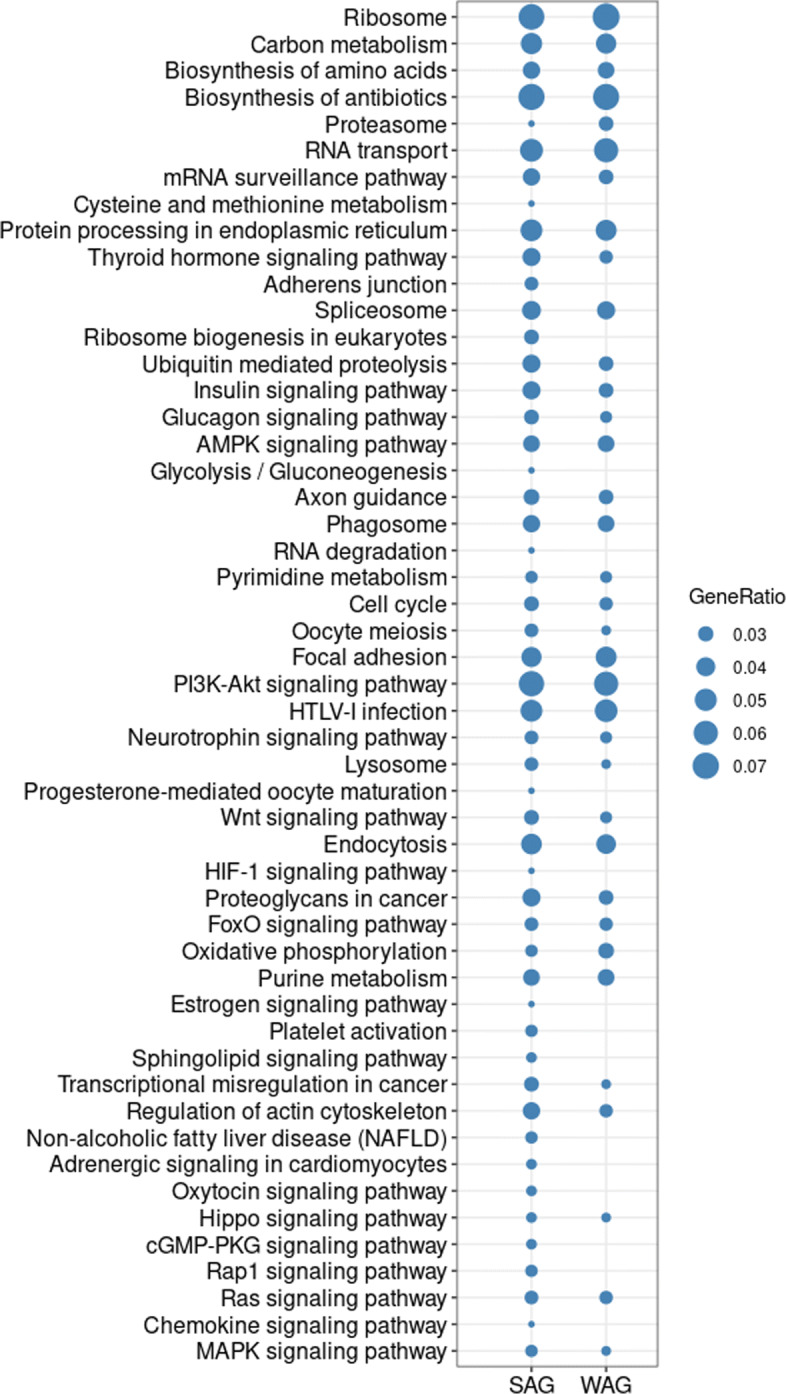
Other pathways involved in miRNA regulation in SAG and WAG cancers

When including all cancer samples regardless of stage into the comparison, 1,147 common miRNA-gene interactions and 51 common critical pathways were identified (Supplementary Table 2). Taking two signaling pathways as examples: 193, 19, and 4 miRNAs regulate MAPK pathways in obesity, liver cancer and uterine cancer, respectively. As shown in Fig. 3A, miR-423-5p and miR-484 are the common regulator in all three diseases by targeting overlapped sets of genes (including NFKB2, GNG12, MKNK2, STMN1, FLNA) in these diseases. Similarly, in PI3K-Akt signaling pathway (Fig. 3B), miR-484 and miR-769-5p co-regulate different genes in three diseases. All these observations stressed again the modulated property of miRNA regulation as different miRNAs can regulate the similar processes by targeting the same or different genes. An expanded list of pathways revealed by the common interactions is shown in Fig. 4.

Additionally, 10 interactions were involved in the non-alcoholic fatty liver disease (NAFLD) pathway, including miR-423-3p, miR-484, miR-342-3p, miR-146b-5p, miR-193a-5p, miR-324-3p and their 10 targets. We used a set of in-house (small) RNA-Seq data generated on a liver disease mouse model (details can be found in Supplementary Materials) and examined the expression patterns related to these interactions. We found that in the mouse liver samples collected at 20 wks (with STZ+HFD induced tumor), the ITCH/miR-324-3p interaction shows negatively correlated expression between miRNA and gene (|Pearson correlation coefficient (PCC)|≥0.7) while RXRA/miR-423-3p, JUN/miR-342-3p, and AKT2/miR-423-3p show weak negative correlation. Similarly, among all 721 LIHC interactions identified in the TCGA data, 328 of them show similar negatively correlated expression (Supplementary Table 3). This coupled result provides clear indication of miRNA participation in cell proliferation, differentiation, and inflammatory signaling during the progression of non-alcoholic fatty liver disease to cancer.

## Discussion

In this study, we identified miRNA-gene interactions in obesity and seven major types of cancer by integrating multi-level genomic information through computational models. A list of common miRNA regulators are highly likely to be involved in the development of obesity-associated cancers in terms of growth and inflammatory signaling and metabolism. Strong evidences show that most miRNAs contribute in the same functional pathway through regulating different genes under different conditions, which indicates that miRNA regulation is a function-driven dynamic process.

We also observe the patterns among the altered functional pathways which are promising to differentiate two groups of obesity related cancers, particularly in the early stage. Note that when including all cancers regardless of stage, due to the much higher variability among the expression profiles, the detected interactions and enriched pathways are slightly different from the early stage cases. This is a common problem in disease-related expression analysis given the fact that many genes and miRNAs show differential expression in different disease stages in addition to the variability related to gender, age, and other factors. Based on the identified conditional miRNA-gene interactions in each disease, we observed that miRNAs regulate a few common signaling and metabolic pathways between obesity and cancers, which may imply the miR-driven linkage between these diseases. Obesity comes from a chronic imbalance between energy intake and energy expenditure, which results in changes that lead to abnormal growth. The cellular location of these changes, which potentially includes the regulation, signaling, and genomic and epigenomic systems, are reflected at the metabolic level. Therefore, metabolic abnormality as one of major hallmark of cancer also reflects the early change involved in the disease development. We believe by focusing on the metabolic aspect of disease progression, we can further study how external factors affect disease progression and understand the association between obesity and cancers at the systems level where miRNA regulation represents a key mechanism in terms of signaling from adipose tissues to remote tissues. We agree that experimental developments along the research line of miRNA tracking and targeting can help better understand miRNA’s involvement in cell communication and disease progression.

It is noted that miRNA transfer and cross-talk is beyond the scope of this computational study due to the difficulty in stratifying miRNAs according to their origins. There are well-known technical challenges in designing experimental protocols to track miRNA secretion and isolate exogenous regulation in target tissues.

From a technical perspective, we have developed a new integrated framework to study dynamic miRNA-mediated regulation in human diseases and have demonstrated that such data-driven approaches with novel solutions to information fusion and computational modeling can effectively facilitate novel mechanistic discoveries and hypothesis testing in biomedical research. The comparison with other methods of the same kind has demonstrated advantageous performance of this methodology (Supplementary materials). In the meantime, we are also well aware of the challenges in data integration from heterogeneous resources. For instance, miRNA-mRNA interactions are deemed to be context-dependent and somewhat cell specific. Ideally, various levels of genetic and genomic data should be collected from the same context to avoid biased discovery. In this study, although all common interactions inferred in obesity and cancers can be validated in miRecords and mirTarBase, this prediction doesn’t include interactions known in obesity and cancer linkage. This is mainly because some miRNAs (e.g., miR-10b, 302b, or -498 in obesity-breast cancer case) were not initially covered in the CLASH interactome data. This issue can be possibly addressed by compiling more complete interactome available in different cell types. For the same reason, it is also problematic to combine or compare different studies when variant context are presented, which may partially explain the current inconsistency of miRNA regulators reported in a disease by different studies in the literature. However, a higher level of conservation in terms of miRNA regulated pathways is expected in those scenarios. Furthermore, existing interactome detected in very few cell types share high level of commonality but as a whole, it is still infeasible to cover all the possible interaction patterns. To this end, future studies with well-thought out experimental design and more systematic data generation can dramatically improve the capacity of similar computational models.

## Conclusion

In this paper, we have examined the association between obesity and obesity-associated cancer through studying the miRNA regulation. Particularly, a novel statistical method has been developed to discover the context-dependent miRNA-gene regulation and identify key miRNA regulators that are involved in the development of obesity-associated cancer. The entire framework for the detection of condition-dependent miRNA-mRNA interaction can be integrated into general dynamic gene regulation network study and be applied in similar biomedical research.

## Supplementary Information

Additional file 1: **Supplementary Materials**.

## Data Availability

The data source information and the link to download the program are provided in the manuscript. A datasets used in this study are summarized in Table 1.

## Abbreviations

CNV: Copy number variation
DE: Differentially expressed
ENCODE: Encyclopedia of DNA elements
FWL: Frisch–waugh–lovell
GEO: Gene expression omnibus
KIRC: Kidney renal clear cell carcinoma
LIHC: Liver hepatocellular carcinoma
LUSC: Lung squamous cell carcinoma
MAPK: Mitogen-activated protein kinases
MFE: Minimum free energy
RS: Regulatory score
SAG: Strong association group
TCGA: The cancer genome atlas
TF: Transcription factor
TSS: Transcription start site
WAG: Weak association group
