# Supplementary material for: Elucidation of molecular links between obesity and cancer through microRNA regulation
Source: BMC Med Genomics. 2020 Oct 30;13:161. doi: 10.1186/s12920-020-00797-8 (PMC7596939; doi:10.1186/s12920-020-00797-8)
Supplement: Supplementary file 1 — Supplementary Materials. [file 12920_2020_797_MOESM1_ESM.pdf]

Supplementary Materials

Animal model

Pathogen-free 14-day pregnant C57BL/6J mice were purchased from Jackson Lab (USA). NASH–HCC was induced in male mice by a single subcutaneous injection of 200 lg STZ (Sigma, MO, USA) at 2 days after birth and feeding with HFD32 (CLEA Japan) ad libitum after 4 weeks of age. Male mice without any treatment, male mice treated with STZ alone and female mice treated with STZ–HFD were housed for control animals.

Liver samples were collected from male mice of five groups (4wks with STZ and then with HFD diet at 6 wks, 8wks, 12 wks and 20 wks) with their corresponding control groups. The number of mice per group per time point for gene and miRNA expression analyses was three, which gives a total of 30 samples.

Transcript and miRNA Sequencing and data analysis

30 liver RNA samples were shipped on dry ice to BGI@CHOP (Philadelphia, PA) for RNA-Seq and small RNA-Seq library preparation and analysis. Specifically, Illumina-HiSeq 4000 platform was used to sequence transcriptome (paired-end 100bp) while the Illumina-HiSeq 4000 platform for small RNA (single-end 50bp). Raw sequence data were deposited in the NCBI SAR database (SUB7793657, official ID pending). Read quality control checks were performed using FastQC [Babraham, 2017]. Adaptors, primers, ambiguous bases, and bases having quality scores less than 30 were removed by cutadapt [Martin, 2011].

RNA-Seq analysis was performed using RSEM and EBSeq for gene expression quantification. For miRNA, high quality reads in the range of 18 - 40 bps remained for annotation and expression estimation using miRDeep2, a small RNA sequencing analytical pipeline [Friedlander, 2012]. Particularly, the read sequences were mapped to reference sequences of mouse genome, known precursor and mature miRNA, and other types of non-coding RNAs. The miRNA expression levels identified for each library were normalized as transcripts per million (TPM) according to the following formula: TPM = (specific miRNA read count ×1,000,000) / total miRNA mapped reads.

Identification of differentially expressed miRNAs and Genes

For the pairwise comparison, *DESeq2* package was used to identify the differentially expressed (DE)- miRNAs and genes in each condition group versus respective control. A threshold of fold change ≥ 2 and P value ≤ 0.05 was employed to identify DE-gene and miRNAs. The table below shows the statistics from this analysis.

	DE	Genes	miRNAs
4wks	Up	384	23
	Down	612	79
6wks	Up	11	35
	Down	16	31
8wks	Up	701	46
	Down	114	69
12wks	Up	432	19
	Down	72	57
20wks	Up	652	70
	Down	201	108

Comparison with

We have conducted a peer comparison with two other well-established methods, RACER[1] and MCMG[2], on miRNA target prediction using expression data, to demonstrate the performance of our approach. The test dataset was collected from 332 Hepatocellular carcinoma (LIHC) patients, available at TCGA[3], along with 34,986 experimental miRNA-target interactions detected by CLEAR-CLIP[4] in Hepatocellular carcinoma cell (Huh-7.5). We utilized three methods to predict the miRNA-target interactions in each stage of LIHC. First, we found that RACER and MCMG predicted significant more interactions than miRDR (average number of predicted interactions: miRDR 1,634; RACER 10,371; MCMG 212,171) but less experimental validated interactions among the top ranked ones. When taking a close look at each stage, we ranked the predicted interactions from RACER and MCMG according to the score and compare with top-n predictions from miRDR at 100-intervals (Figure 1A). It is clear that miRDR outperformed two other methods by identifying significantly more validated interactions in each stage (p-value of one-sided Wilcoxon singed rank test is shown in the figure). Moreover, unlike RACER and MCMG, miRDR's performance was not affected by the input sample size. For example, RACER and MCMG predicted only half validated targets in smaller sets (in stage II (82), III (80) and IV (6)) than what they did in a bigger set (stage I (164)). At another evaluation, we compared the distribution of miRNA target count predicted by three methods, using the experimental data from CLEAR-CLIP as reference. As Figure 1B shows, the distribution of miRDR is very close to the experimental one, while RACER and MCMG tend to predict too many more targets for most miRNAs.

(1. Li, Y., Liang, M., Zhang, Z., Luo, J. & Zhang, Z. Regression analysis of combined gene expression regulation in acute myeloid leukemia. PLoS Comput Biol 10, e1003908 (2014).; 2. Chen, X., Slack, F. J. & Zhao, H. Joint analysis of expression profiles from multiple cancers improves the identification of microRNA-gene interactions. Bioinformatics 29, 2137–2145 (2013). ; 3. Ally, A. et al. Comprehensive and Integrative Genomic Characterization of Hepatocellular Carcinoma. Cell 169, 1327–1341.e23 (2017). ; 4. Moore, M. J. et al. miRNA-target chimeras reveal miRNA 3'-end pairing as a major determinant of Argonaute target specificity. Nat. Commun. 6, 8864 (2015))

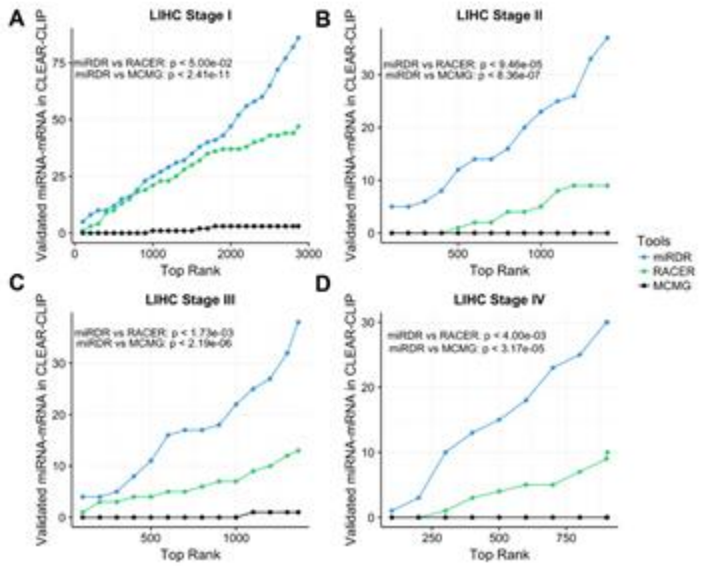

Figure 1A. Identified miRNA-target interactions identified by MiRDR, RACER and MCMG based on the Hepatocellular carcinoma (LIHC) data. The x-axis represents the top ranked interaction predicted by miRDR while the y-axis represents the count of validated interactions. The p-values were calculated by one-sided Wilcoxon singed rank test.

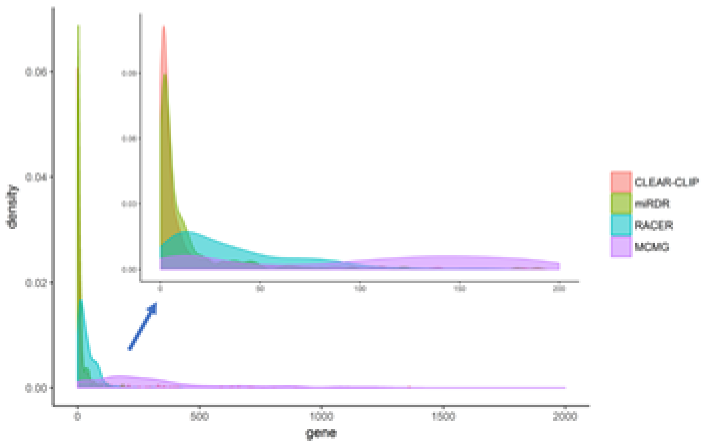

Figure 1B. Method comparison based on the counts of identified miRNA targets. X-axis: number of target genes; y-axis: miRNA percentage. Outer plot shows the overall distribution and the inner shows the zoom-in view of partial distribution in the range of [0, 200]

Through a comparison with two leading algorithms for miRNA target prediction based on expression profiles, miRDR has showed its advantageous predictive power with two major improvements. First, comparing with other methods, miRDR's predictions tend to include more validated interactions while not introducing significant high false prediction. This is an important aspect given the widely accepted critique on high false positive rate in current target prediction. Another advantage was the stable performance of miRDR on fairly limited sample size. The meta-analysis design minimized the impacts of sample variation on the target prediction, which seems promising to facilitate the detection of weak signals between miRNA and targets with insufficient samples.

Supplementary Table 1. The summary of differentially expressed genes and miRNAs, as well as miRNA-gene interactions detected in each disease condition.

	Obsity	KIRC	KIRP	LIHC	UCEC	BRCA	LUAD	LUSC
DE-Gene	405	4944	4660	3696	5599	4231	4426	6365
DE-miRNA	9	72	72	79	144	69	73	97
Detected gene-miRNA interactions	9,123	475	805	813	301	1,011	800	718

(The detailed list is provided at http://sbbi-panda.unl.edu/misc/obesity_prj_diff_genes.zip; the list is already filtered according to $|\log_2 FC| <= 1$ and pval <= 0.05)

Supplementary Table 2. Common miRNA-gene regulatory interactions and pathways between obesity and SAG cancer.

**Common interactions between obesity and cancers of Strong Association Group**	**miR-484** **(361)** - ABCD4/ABHD12/ABHD2/ACADS/ACLY/ACTA2/ACTB/ADAM10/ADCK2/ADRM1/AGK/AGL/AIFM1/AKAP13/AKT1S1/ALAS1/ALKBH5/AMMECR1L/AMPD2/ANAPC1/ANXA11/AP1B1/AP1G1/AP2M1/APLP1/ARAP1/ARFGEF2/ARHGDIA/ARMC6/ATN1/ATP1A1/ATXN7L3/AUP1/AXIN1/BACE2/BAG5/BAMBI/BCAT2/BCL2L1/BLMH/BMS1/BRI3/C1orf21/CAPRIN1/CASC3/CBX5/CBX6/CCDC47/CCNK/CD2AP/CDC34/CDCA7L/CDS2/CHAF1B/CHD4/CHMP7/CHSY3/CHTF18/CLDN7/CNFN/CNIH4/COX8A/CPSF7/CRTAP/CUL5/CUTA/CYB5R3/CYFIP1/DCTN3/DCTN5/DDX28/DDX41/DDX54/DDX6/DGAT1/DGCR2/DGCR6L/DHX40/DIP2A/DLG5/DNAJA3/DNASE1L1/DNMT1/DST/DVL2/DYRK2/EEF2/EFNB3/EIF4B/EIF5B/EIF6/EMD/ENOPH1/ENTPD4/EXOC6B/EXOG/EXOSC10/EZH2/FAM117A/FAM160B2/FBRS/FBXL18/FBXW2/FGFBP3/FIZ1/FKBP4/FLII/FLOT1/FURIN/FUT11/FUZ/FYCO1/GANAB/GAPDH/GATAD2B/GEMIN4/GFOD1/GHITM/GIGYF1/GIT1/GNL1/GNL2/GOLM1/GOT2/GPAM/GPATCH4/GRPEL1/GTF2H3/GTF2H4/GTF3C1/HARS2/HCFC1/HECTD3/HIC2/HIPK1/HK2/HMGXB3/HOMER1/HSD17B4/HSP90AB1/HSPBP1/HYOU1/IFNAR2/IGF1R/INTS5/IPO5/IRS2/ISM2/ITGA5/ITGA9/IVD/JAG2/KDM5C/KIAA1109/KLC2/KNTC1/KREMEN1/L3MBTL2/LAMP1/LBR/LETM1/LMNB1/LONRF2/LRRC20/LSM12/LUC7L3/M6PR/MAF1/MAN2A2/MAP3K10/MAP7D1/MAST3/MCM3AP/MCM5/MCM7/MDH2/MDM2/MDN1/MEGF8/METTL2A/MLH3/MLLT1/MSH6/MSI2/MSL3/MTCH2/MTHFD1L/MTR/MYH9/MYO10/MYO5A/MYO9B/NCAPH/NCOR1/NDST1/NDUFV1/NEK1/NEO1/NFE2L1/NMD3/NME4/NOC2L/NOM1/NOMO3/NOTCH3/NPEPPS/NRIP2/NUCKS1/NUDC/NUS1/OCRL/ORAI2/P4HB/PDE12/PDLIM2/PER1/PGAM1/PHB/PHF23/PHKA1/PHKB/PHLPP1/PHTF1/PI4KA/PIN1/PKD1/PLD3/PLOD1/PLXNA1/POLDIP3/POLR3K/POM121/PPP2R5A/PRDX1/PRKCD/PRMT7/PRPF8/PSMC2/PSMC3IP/PSMC5/PTGR2/PTRH2/PUF60/PYGB/RAB40C/RBM39/RBM42/RBMS2/RC3H2/RCC2/RCE1/RER1/REXO1/RGP1/RNF167/RNF5/RNMT/RPL21/RPL28/RPL36/RPN2/RPS14/RPS18/RPS24/RPS9/RRAGD/RUNX2/RXRB/SAP18/SCAP/SEC11A/SEMA4D/SEPHS1/SESN2/SETD7/SF3B2/SF3B3/SH2B1/SLC10A7/SLC11A2/SLC19A1/SLC2A1/SLC38A1/SLC39A13/SLC43A1/SMAD2/SNRPD2/SNX11/SORD/SPIRE1/STIP1/STK10/STMN1/STRN/SUPT16H/SYNE2/SYNGR2/TBC1D15/TBC1D22A/TBC1D2B/TBRG1/TCF19/TEP1/TEX261/TFAM/TIMELESS/TIPIN/TKT/TLN1/TMED9/TMEM184B/TMEM50A/TMOD2/TNFRSF12A/TNK2/TNKS1BP1/TNRC6B/TOMM40/TPST2/TRAF7/TRIM28/TRIM65/TROAP/TSC22D3/TSEN54/TSPAN3/TSPAN33/TSPAN6/TTYH3/TUBB/TUBB2A/TUBGCP4/TULP4/TUT1/TWF2/TXLNA/UBE2Z/UBQLN1/UBQLN2/UBQLN4/URGCP/USP40/WAC/WDR26/WDR4/WEE1/YBX1/YWHAZ/ZC3H7B/ZFX/ZFYVE21/ZNF121/ZNF202/ZNF226/ZNF264/ZNF362/ZNF383/ZNF48/ZNF792<br><br>**miR-331-3p** **(137)** - ACTR1A/ANKRD52/AP2A2/APBB1/ARAF/ARHGEF1/ATP8B2/ATRX/AURKAIP1/BOK/BRPF1/C10orf88/C12orf65/CAMK2G/CDCA4/COASY/COCH/COPS3/COX7C/CTNNB1/CUEDC2/DDX56/DEF8/DGKZ/DHX8/DMAP1/EARS2/EFNB2/EIF2S3/EIF4A2/EIF4A3/EIF4G2/EPB41L2/EPHA7/EXOC6B/EXOC7/FAM168B/FASN/FASTK/FBXL5/FBXO44/FLII/FLT4/FUS/GATAD2B/GNAS/GNPAT/GPI/GSS/GTF3C4/HNRNPH1

	/HPS1/HSPD1/IGF2BP2/IGFBP5/IL4R/KLC2/LETM1/MAP4/MARK3/MECP2/MED22/MNT/MRPS26/MYBBP1A/NCLN/NDST1/NRBF2/NXT1/PCBD1/PHGDH/PHLDB3/PI4KB/PINX1/PITX1/PLCH2/PLXNB2/POLR2L/POLR3H/PPARD/PPIL1/PSMC4/PSMD6/PSME3/PTPN13/RANGAP1/RASL10B/RDX/RNASEH1/RNF145/RPL34/RPL7A/RPS12/RPS14/RPS9/RRBP1/RRP1B/RRP7A/SCD/SF3B2/SFPQ/SH3BP2/SH3PXD2A/SLC25A14/SLC39A8/SMG5/SMG6/SMO/SMPD4/SPTB/SS18/SSR4/STK35/STX16/SUV39H1/SYK/SYNM/TIA1/TJAP1/TOMM20/TRAP1/TSFM/UBA1/UBE2Q2/UBQLN1/VAPB/VPS37B/WASH3P/XRCC5/YTHDF1/ZNF483/ZNF503/ZNF512B/ZNF598/ZNF607/ZNF608/ZNF714<br><br>**miR-324-5p (108) -**<br>ACTN4/AGAP1/AHNAK/ANAPC13/ANXA6/ANXA7/ARFGAP2/ARHGEF10L/ARPP19/ASF1B/ASS1/BOP1/BTBD2/BTN3A1/CACHD1/CBS/CDC123/CDS1/CENPB/CLN6/CLPB/COL14A1/COL6A1/COPS3/COX5A/CUEDC2/DDX11/DYRK1A/EDC3/ESD/FAM43B/FAM83H/FBL/FOXC1/GIGYF1/GMNN/GNB2/HMGXB3/HNRNPA1/HSPD1/IPO5/ITM2C/KIF3B/KLHDC3/LDHB/MAPKAPK2/MDC1/MEA1/MID2/MPI/MRPL14/MSH6/MTHFD1/MYO1C/MYOD1/NANS/NDUFS1/NUP160/OSTC/PA2G4/PACS1/PAK4/PARM1/PDRG1/PGAM1/PGK1/PIAS4/PKD1/PMS2/POLD1/POLR2A/PSMA4/PSMD4/PTK7/PXDN/RAB1B/RAP2B/RAPH1/RPL27/RPL37/RPP30/RPS2/SAP30BP/SCARB1/SCD/SCD5/SEC23IP/SLC25A3/SMAD2/SMARCD2/SNX7/SOBP/SPEN/SPOPL/TBCB/TIMELESS/TMEM129/TMEM63B/TMEM8B/TRERF1/TUBA1B/TUBA1C/TUFM/UBE2I/USP16/WIZ/ZIC5/ZMYND11<br><br>**miR-615-3p (75) -**<br>ABT1/ACTN4/ALDH1B1/ANKRD36B/ANO6/ATRX/BCKDK/BICD2/BRD2/BTN3A2/CCND2/CHD4/CTNNA1/CYC1/DBN1/DNM1L/EIF3M/EIF4G1/EPDR1/EPN2/EWSR1/FAM13A/FNBP1/FRAT2/FSTL1/GANAB/GGA3/GIGYF1/GNL3/GPSM1/HDAC7/HRAS/KDM4B/KIAA1109/KIFC2/LIG1/LRPAP1/MAPKAPK2/MATR3/MCM10/MORF4L1/MRPL23/NCAPD2/NOP2/NUPR1/OGFOD1/PA2G4/PABPC4/PDCD11/PDS5A/PFDN6/PKD1/PLXNA1/POLR1A/POLR2B/PTGFRN/PTMA/RARA/RNF25/SCARB1/SEC24C/SEPHS2/SF3B1/SGMS2/SLC1A4/SMC3/SMG1/SOCS7/TPM3/TRPC1/TUBB2B/UBA1/XYLT2/YTHDF2/ZNF687<br><br>**miR-423-3p (69) -**<br>ABHD12/ADCY1/AKT2/ALAS1/APP/ARFGAP1/ATP6V0D1/ATP8B2/B4GALNT3/C1orf56/CALR/CEP78/COPA/CYFIP2/DGCR6L/DPYSL3/ETF1/FADS2/FASN/FEM1A/FLII/FOXJ2/FRMD4A/GLDC/HDLBP/IER3IP1/IGF2R/KDELR1/KIAA0319L/LARP1B/LARP4/LRRC8B/LSM4/LYPLA2/MAP3K1/MCM2/MOGS/MTHFR/NPTX2/NTN1/NUBP1/PEPD/POLR2L/POM121/PPP2R1B/PSMD1/PSMD8/PTCD2/PTMS/RANBP2/RBBP4/REEP2/ROBO1/RPL3/RPL4/SCML2/SET/SH2B1/SHMT2/SLC25A15/SMARCD2/TAGLN2/TBCE/TXLNA/UBE4B/URB2/WNK1/YIPF5/ZNF324<br><br>**miR-423-5p (59) -**<br>ABCC5/AKAP13/ARHGAP32/ARL6IP1/ATN1/ATP6V1E1/BCCIP/CCNF/CD81/CHD8/DDX21/DDX24/EPM2AIP1/FAM83H/FAM98A/FHOD1/FKBP4/GDF11/GGA3/GNG12/KCTD15/KCTD2/LMNB2/LRCH4/MED28/MFGE8/MICALL1/MIER2/MRPL34/NECAP1/NFKB2/NMD3/NOS1AP/NPLOC4/NSD1/PAX2/PCBP1/PDPK1/PLCB1/PMPCA/PTTG1/RABAC1/RC3H1/RCE1/RPS15A/SALL1/SAR1B/SF3B3/SLC7A2/SLC9A3R1/SQSTM1/TDRKH/TMUB1/UBE2L3/ULK1/USP6/VAC14/WBP2/YWHAZ<br><br>**miR-455-3p (57) -**<br>AGL/AP1B1/ATP6AP1/BAIAP2/BCL2L12/BRD4/BTF3/CISD2/CLSTN1/COL3A1/CRIM1/DAZAP2/DDX55/DYNC1H1/EIF2B2/EIF4G1/ELOVL1/EPHA7/ERI3/EVC/FDX1/GALM/GBA2/GIGYF2/HMGN2/HYOU1/INTS10/INTS4/LRP11/LYRM1/MAEA/MAP1B/MGRN1/N4BP2L2/NPTN/PFKP/PGM3/PGRMC1/PLEKHM2/PTPRF/PWP2/RANBP3/RHOC/RNF216/RPS5/RRP1B/SAT1/SCD/STX16/TFE3/TNRC18/TOR1AIP2/U2AF2/USP10/USP5/WRAP53/XPR1<br><br>**miR-324-3p (42) -**<br>ACACA/ANKRD27/ARHGAP17/ATXN2L/BAHD1/CAPZB/COX7A2L/CSNK1A1/CXXC1/CXXC5/DDX18/DLST/EEF2/FADS2/GAPDH/HSP90AA1/HSPA9/HUWE1/IQGAP1/ITCH/KDM2B/MED15/NHP2/PNMA2/POLR2C/PPFIA1/PPIE/PRKAR1A/PUM1/RPL10A/RPL31/RPLP0/RPS3/RPS5/RPS8/RUVBL2/SAP18/SBF1/SMS/TMED9/TRAK1/VPS18<br><br>**miR-125a-5p (38) -**<br>ATXN7L3/CASC4/CGNL1/CLCN7/COL4A1/DLST/EMID1/FBXO10/IP6K1/KIAA0232/LAMB1/MACF1/MAP3K1/MAT2A/MORC2/MRPL50/NNT/OTUB2/PAM/PDCL3/PEG10/PGM3/PLS3/PPP2R5E/PRC1/PREPL/RPS2/SLFN11/TBC1D1/TFRC/THRAP3/TPM4/UBE2G2/XPO1/YES1/YIPF4/ZDHHC9/ZMYND19<br><br>**miR-342-3p (35) -**<br>ABL1/ACTG1/ARIH2/CBX1/CCDC6/CRB2/EEF2/FIBP/FIGN/FN3KRP/GART/GJA1/GOT2/HNRNPC/IDH3B/IDS/INO80D/KCTD15/KPNA2/METTL2A/NAPG/NOP2/PRKCSH/PSAT1/PWP2/RPL26/RPL27A/RPS3A/RRM2/SLC30A9/TACC1/TCP1/TRERF1/VPS35/XPOT

	**miR-769-5p (30)** - ADPGK/AK1/ATP11A/CNOT6/CREBBP/DDX24/DPH5/EIF5A/FANCD2/FBXO45/FKRP/GOT1/GPCPD1/IGSF3/LPIN2/MAPK1/NBPF15/PHLPP2/PLEKHA1/PRDX4/PRUNE2/QRSL1/RNF40/RPL14/SCML2/SPAG5/TMEM165/TRMT61A/UBQLN4/WDR18<br><br>**miR-361-5p (27)** - ACLY/ATP6V1A/BSG/CNOT2/CYCS/DCTN1/DOCK6/EGFL7/GRK6/GSPT1/LNPEP/MAGEF1/MDC1/PPA1/PRPF8/PSMD2/RAVER1/RPS2/SETD1A/SLC25A5/SLC30A5/TMEM62/TP53BP1/UCK2/VBP1/VCP/WBP2<br><br>**miR-339-5p (14)** - ABCC1/AKAP10/ANKRD52/BRSK1/HSPA12A/MAP4/NCSTN/PAXIP1/PGD/PSAP/SMC1A/SOX11/SSR2/SUCLG1<br><br>**miR-671-5p (13)** - C17orf58/CHPF2/CRLF3/CRTC3/GPI/LGALS3BP/MOSPD3/NACA/NRSN2/R3HDM2/RPL23A/U2AF2/XRCC6<br><br>**miR-28-5p (10)** - AAAS/CENPV/CS/GEMIN4/IMPDH1/KLHL12/SIK1/TUFM/TULP4/ZBTB47<br><br>**miR-330-5p (8)** - BAD/CARD11/FTSJ3/NHP2/POMGNT1/SPTBN2/STX1B/TUFT1<br><br>**miR-532-3p (8)** - AFF4/CRY2/EIF3I/KLHL15/MED14/PPP3R1/SBK1/SCMH1<br><br>**miR-107 (5)** - DHX16/GNS/TCF19/TMEM87A/ZADH2<br><br>**miR-28-3p (5)** - CYB5B/HIC2/INTS2/SNX1/VPS53<br><br>**miR-501-5p (5)** - GAPDH/HSP90AA1/HSP90AB1/RPS19/TAF15<br><br>**miR-502-3p (5)** - ASXL2/COMMD4/DCTN2/MGAT5/PITRM1<br><br>**miR-146b-5p (4)** - AKT3/NSFL1C/PLA2G4A/XPO4<br><br>**miR-193a-3p (3)** - CPSF2/SEMA3D/SNRPB2 **miR-320b (3)** - EPB41/GNAS/PPIA<br><br>**miR-330-3p (3)** - PIK3R4/SLIT2/SNRPD3 **miR-361-3p (3)** - CIAPIN1/NFATC3/PRMT3<br><br>**miR-140-3p (2)** - AIFM2/FAU **miR-140-5p (2)** - CASD1/GATA6 **miR-188-5p (2)** - ABCC5/SBNO1 **miR-3200-3p (2)** - DDB1/TPT1 **miR-342-5p (2)** - DDX1/SMARCA4<br><br>**miR-362-5p (2)** - NOMO3/TMF1 **miR-501-3p (2)** - DENND4B/MCM2 **miR-193a-5p (1)** - ZC3H7B **miR-326 (1)** - PBX2 **miR-3615 (1)** - NIPA2 **miR-455-5p (1)** - TRIM23 **miR-574-3p (1)** - MKRN1 **miR-576-5p (1)** - PNMA2
**Enriched pathways based on common interactions**	Adherens junction, Adrenergic signaling in cardiomyocytes, AMPK signaling pathway, Axon guidance, Biosynthesis of amino acids, Biosynthesis of antibiotics, Carbon metabolism, Cell cycle, cGMP-PKG signaling pathway, Chemokine signaling pathway, Cysteine and methionine metabolism, Endocytosis, Estrogen signaling pathway, Focal adhesion, FoxO signaling pathway, Glucagon signaling pathway, Glycolysis / Gluconeogenesis, HIF-1 signaling pathway, Hippo signaling pathway, HTLV-I infection, Insulin signaling pathway, Lysosome, MAPK signaling pathway, mRNA surveillance pathway, Neurotrophin signaling pathway, Non-alcoholic fatty liver disease (NAFLD), Oocyte meiosis, Oxidative phosphorylation, Oxytocin signaling pathway, Phagosome, PI3K-Akt signaling pathway, Platelet activation, Progesterone-

	mediated oocyte maturation, Proteasome, Protein processing in endoplasmic reticulum, Proteoglycans in cancer, Purine metabolism,<br><br>Pyrimidine metabolism, Rap1 signaling pathway, Ras signaling pathway,<br><br>Regulation of actin cytoskeleton, Ribosome, Ribosome biogenesis in eukaryotes, RNA degradation, RNA transport, Sphingolipid signaling pathway, Spliceosome, Thyroid hormone signaling pathway, Transcriptional misregulation in cancer, Ubiquitin mediated proteolysis, Wnt signaling pathway

Supplementary Table 3. Expression correlation between gene and miRNA in the A) NAFLD-related interactions and B) LIHD related interactions, in the mouse liver samples

A)

Gene	miRNA	Expression correlation (PCC) at 20wks
ITCH	miR-324-3p	-0.70315
RXRA	miR-423-3p	-0.33742
JUN	miR-342-3p	-0.25494
AKT2	miR-423-3p	-0.22478
MLX	miR-484	-0.02209
CASP7	miR-484	0.098137
SREBF1	miR-484	0.142232
CYCS	miR-361-5p	0.168155
CEBPA	miR-193a-5p	0.449619
AKT3	miR-146b-5p	0.578952
IRS2	miR-484	0.843737

B)

Gene	miRNA	Expression correlation (PCC) At 20wks
NDUFV1	miR-484	-0.97611
SMS	miR-324-3p	-0.95962
DEAF1	miR-324-5p	-0.95048
ANXA11	miR-484	-0.94884
COL3A1	miR-455-3p	-0.94040
APTX	miR-342-3p	-0.93215

TRAP1	miR-342-3p	-0.93144
PHLDB3	miR-331-3p	-0.92962
DCTN1	miR-361-5p	-0.92215
TFAM	miR-484	-0.92074
YWHAZ	miR-455-3p	-0.91321
PRDX1	miR-484	-0.91157
NUDC	miR-484	-0.89824
ARL6IP4	miR-484	-0.89722
RER1	miR-484	-0.89235
RAB3IP	miR-484	-0.87295
RHOC	miR-455-3p	-0.87010
GRPEL1	miR-484	-0.86690
BRI3	miR-484	-0.86627
TRAP1	miR-324-5p	-0.86528
CBX5	miR-484	-0.86513
PPARGC1B	miR-484	-0.86033
SORD	miR-484	-0.85959
SYNGR2	miR-484	-0.85806
FBL	miR-324-5p	-0.85619
AUP1	miR-484	-0.85482
MKNK2	miR-423-5p	-0.85426
MAP1B	miR-455-3p	-0.85392
MRPS24	miR-484	-0.85125
RAB40C	miR-484	-0.84929
SEPHS1	miR-484	-0.84750
RING1	miR-484	-0.84408
UBA2	miR-330-5p	-0.84337
PWP2	miR-455-3p	-0.84240

MAEA	miR-455-3p	-0.84131
RRP1B	miR-455-3p	-0.83698
PPP2R5A	miR-484	-0.83675
BAMBI	miR-484	-0.83125
DGAT1	miR-484	-0.83069
MARK3	miR-331-3p	-0.82721
COX5A	miR-484	-0.82492
DCTN3	miR-484	-0.82425
ULK1	miR-423-5p	-0.82201
FUT11	miR-331-3p	-0.82053
MTA2	miR-423-3p	-0.81762
RPLP2	miR-484	-0.81236
IDH3B	miR-342-3p	-0.80804
TMEM62	miR-361-5p	-0.80579
ATL2	miR-484	-0.80245
CCT3	miR-330-5p	-0.80114
CDC34	miR-484	-0.79869
SMARCD2	miR-324-5p	-0.79208
COL4A1	miR-125a-5p	-0.79186
HSPA1B	miR-324-5p	-0.79126
TYMS	miR-484	-0.78939
FASTK	miR-331-3p	-0.78821
TCP1	miR-342-3p	-0.78739
AP3M1	miR-423-5p	-0.78660
COX8A	miR-484	-0.78476
ATXN2L	miR-324-3p	-0.77795
RPL27A	miR-342-3p	-0.77223
ECHDC2	miR-484	-0.76793

GEMIN4	miR-484	-0.76426
PFKP	miR-455-3p	-0.76299
MRPL14	miR-324-5p	-0.75299
LYRM1	miR-455-3p	-0.74094
FBXW2	miR-484	-0.73828
CCNK	miR-484	-0.73766
POLR2L	miR-331-3p	-0.73537
PSMD4	miR-324-5p	-0.73373
RPL26	miR-342-3p	-0.73322
NSD1	miR-423-5p	-0.73230
TRPM2	miR-423-3p	-0.72938
RPS3	miR-324-3p	-0.72320
POLR2E	miR-339-5p	-0.72264
DNAJC11	miR-324-5p	-0.72025
LRP11	miR-455-3p	-0.71622
BSG	miR-361-5p	-0.71588
ABCD4	miR-484	-0.70742
ITCH	miR-324-3p	-0.70315
SOX11	miR-339-5p	-0.70031
YBX1	miR-484	-0.69785
MGRN1	miR-455-3p	-0.69716
TEX261	miR-484	-0.69631
BRSK1	miR-339-5p	-0.69567
TBC1D1	miR-125a-5p	-0.69453
SHCBP1	miR-455-3p	-0.69392
RIOK2	miR-484	-0.68872
UBC	miR-324-3p	-0.68741
CLSTN1	miR-455-3p	-0.68560

DBT	miR-324-5p	-0.68127
TMEM50A	miR-484	-0.67466
NXT1	miR-331-3p	-0.67371
FOXC1	miR-324-5p	-0.66937
USPL1	miR-324-5p	-0.66723
ZNF512B	miR-331-3p	-0.66542
RANBP2	miR-423-3p	-0.66498
ATAD1	miR-146b-5p	-0.66341
POM121	miR-423-3p	-0.65999
ABCC1	miR-339-5p	-0.65957
FAU	miR-140-3p	-0.65087
RNF167	miR-484	-0.64508
GIGYF1	miR-484	-0.64451
PHF23	miR-484	-0.64324
CASC3	miR-484	-0.64292
WBP2	miR-423-5p	-0.64172
CEBPB	miR-423-5p	-0.63724
EIF2B2	miR-455-3p	-0.63676
PLEKHM2	miR-455-3p	-0.63518
POLR2A	miR-484	-0.62776
TXLNA	miR-423-3p	-0.62209
LBX2	miR-339-5p	-0.62146
ADCK2	miR-484	-0.61694
CLPB	miR-324-5p	-0.61412
CDCA4	miR-331-3p	-0.61397
UNC45A	miR-125a-5p	-0.61376
SEC11A	miR-484	-0.60559
LARP4	miR-423-3p	-0.60402

ZIC5	miR-324-5p	-0.60385
SYNM	miR-331-3p	-0.60352
NUCKS1	miR-484	-0.60016
YY1	miR-484	-0.59993
NOC2L	miR-484	-0.59993
KCTD15	miR-423-5p	-0.59955
LNPEP	miR-361-5p	-0.59124
PHB	miR-484	-0.58931
AURKAIP1	miR-331-3p	-0.57772
AIFM1	miR-484	-0.56857
TUFM	miR-324-5p	-0.56614
LUC7L3	miR-484	-0.56510
RC3H2	miR-484	-0.56470
COX7C	miR-331-3p	-0.55879
PTP4A1	miR-324-3p	-0.55714
RPL4	miR-423-3p	-0.55462
SBF1	miR-324-3p	-0.55358
SMG5	miR-331-3p	-0.55261
SRCAP	miR-484	-0.54966
SDF4	miR-484	-0.54961
BCL9L	miR-331-3p	-0.53897
LAMP1	miR-484	-0.53707
RRP1B	miR-125a-5p	-0.53095
MAP3K10	miR-484	-0.52723
MTCH2	miR-423-5p	-0.52711
INO80D	miR-342-3p	-0.52103
TPM4	miR-125a-5p	-0.51935
ATXN7L3	miR-484	-0.51240

PGM3	miR-455-3p	-0.51204
SF3B1	miR-423-3p	-0.50653
WDR26	miR-484	-0.50650
TIMM50	miR-324-5p	-0.50584
SLC25A1	miR-324-3p	-0.50359
VBP1	miR-361-5p	-0.50189
MRPL50	miR-125a-5p	-0.49804
PKD1	miR-484	-0.49411
ANAPC13	miR-324-5p	-0.49354
SUV39H1	miR-331-3p	-0.49332
CDC123	miR-324-5p	-0.49281
PTMS	miR-423-3p	-0.49217
PMM2	miR-484	-0.49178
CASD1	miR-140-5p	-0.49149
MEA1	miR-324-5p	-0.49121
IGFBP5	miR-331-3p	-0.48779
RPL26	miR-423-3p	-0.48035
RPS14	miR-331-3p	-0.47834
POLG	miR-484	-0.47697
GRIPAP1	miR-484	-0.47666
TRMT61B	miR-331-3p	-0.47596
RPLP0	miR-331-3p	-0.47520
WNK3	miR-125a-5p	-0.47178
GNG12	miR-423-5p	-0.46768
RPL3	miR-423-3p	-0.46069
PDCL	miR-423-3p	-0.45853
CUEDC2	miR-331-3p	-0.45616
STX16	miR-324-3p	-0.45547

STX1B	miR-330-5p	-0.43903
PSMA4	miR-324-5p	-0.43018
MRPS26	miR-331-3p	-0.42804
TOMM40	miR-484	-0.42768
SAP30BP	miR-324-5p	-0.42578
LSM4	miR-423-3p	-0.42165
RPL29	miR-331-3p	-0.40678
NFATC3	miR-361-3p	-0.40306
GIGYF1	miR-324-5p	-0.40251
ISM2	miR-484	-0.40248
ALAS1	miR-484	-0.39889
WEE1	miR-484	-0.38970
MBOAT7	miR-484	-0.38944
PLA2G4F	miR-331-3p	-0.38938
RBM39	miR-484	-0.38242
TMEM129	miR-324-5p	-0.38228
PDE12	miR-484	-0.38221
NDUFS1	miR-324-5p	-0.37608
PAM	miR-125a-5p	-0.37469
ARHGAP26	miR-324-3p	-0.37238
PI4KA	miR-484	-0.37067
POLR2C	miR-324-3p	-0.36692
PKD1	miR-324-5p	-0.36414
ECE1	miR-484	-0.35816
EMID1	miR-125a-5p	-0.35709
PSMC4	miR-331-3p	-0.35673
RAB1B	miR-324-5p	-0.35661
COASY	miR-331-3p	-0.35589

FASN	miR-423-3p	-0.35335
ATN1	miR-484	-0.35195
DNAJA3	miR-484	-0.34756
VASP	miR-330-5p	-0.34621
P4HB	miR-484	-0.34006
WAC	miR-484	-0.33859
RNF216	miR-455-3p	-0.33682
NMD3	miR-484	-0.33629
RPL7A	miR-331-3p	-0.33575
CUEDC2	miR-324-5p	-0.33339
PRDX4	miR-484	-0.33163
ACACA	miR-324-3p	-0.33112
YWHAH	miR-324-5p	-0.33028
RPL34	miR-331-3p	-0.32927
PCNT	miR-484	-0.32679
NME3	miR-324-5p	-0.32526
DMAP1	miR-331-3p	-0.32484
DENND4B	miR-501-3p	-0.32226
SET	miR-423-3p	-0.32145
ACADS	miR-484	-0.31936
PGK1	miR-324-5p	-0.31712
HDLBP	miR-423-3p	-0.31107
CSNK1A1	miR-324-3p	-0.30965
HOMER1	miR-484	-0.30926
STK35	miR-331-3p	-0.30902
TNFAIP8L1	miR-484	-0.29910
NHP2	miR-324-3p	-0.29812
MAP3K1	miR-423-3p	-0.29753

SPOPL	miR-324-5p	-0.28651
KLHL15	miR-423-3p	-0.28583
PSMD8	miR-484	-0.28538
POLR3H	miR-331-3p	-0.28434
TACC1	miR-342-3p	-0.28211
GGA3	miR-423-5p	-0.28021
ELOVL1	miR-455-3p	-0.27644
ADRA1A	miR-484	-0.27116
WIZ	miR-324-5p	-0.26877
PHKB	miR-484	-0.26509
NUS1	miR-484	-0.25889
LETM1	miR-331-3p	-0.25844
APRT	miR-484	-0.25573
PLCB1	miR-423-5p	-0.25372
AP1B1	miR-455-3p	-0.24592
ATP8B2	miR-331-3p	-0.24589
TRIM65	miR-484	-0.24261
DYNC1H1	miR-455-3p	-0.24246
RAVER1	miR-361-5p	-0.22926
BCL2L1	miR-484	-0.22864
SOBP	miR-324-5p	-0.22490
ASCC2	miR-484	-0.22475
ZC3H7B	miR-193a-5p	-0.22316
CAPZB	miR-324-3p	-0.22131
PPA1	miR-361-5p	-0.22103
ABHD12	miR-484	-0.22054
PGM3	miR-125a-5p	-0.21740
NPLOC4	miR-423-5p	-0.21367

NUP188	miR-324-5p	-0.21364
CNOT1	miR-423-3p	-0.21222
ZFYVE27	miR-423-3p	-0.21159
IRAK1	miR-330-5p	-0.20494
GNB2	miR-484	-0.20082
PDPK1	miR-423-5p	-0.19443
SBK1	miR-532-3p	-0.18793
PCBD1	miR-331-3p	-0.18132
DCTN5	miR-193a-3p	-0.18063
NISCH	miR-455-3p	-0.17993
ARRB2	miR-330-5p	-0.17980
ILF3	miR-532-3p	-0.17976
AP2M1	miR-484	-0.17800
ROBO1	miR-423-3p	-0.17252
KCTD15	miR-342-3p	-0.17192
ALAS1	miR-423-3p	-0.17063
TCF3	miR-342-3p	-0.17047
HNRNPUL1	miR-423-3p	-0.17026
SAP18	miR-324-3p	-0.16792
ASF1B	miR-324-5p	-0.16571
HIPK2	miR-423-5p	-0.16529
CALR	miR-423-3p	-0.16513
FAM83H	miR-423-5p	-0.16106
YTHDF1	miR-331-3p	-0.15704
GNS	miR-484	-0.15621
ARFGAP1	miR-423-3p	-0.15217
MECP2	miR-331-3p	-0.13294
AK2	miR-193a-3p	-0.13157

PGRMC1	miR-455-3p	-0.13084
TRIM23	miR-455-5p	-0.13021
SLC25A5	miR-361-5p	-0.12984
KPNA2	miR-342-3p	-0.12923
GANAB	miR-484	-0.12889
SPTB	miR-331-3p	-0.12852
DDX56	miR-331-3p	-0.12773
MTHFR	miR-423-3p	-0.11923
EPM2AIP1	miR-423-5p	-0.11699
PHTF1	miR-484	-0.11684
HUWE1	miR-324-3p	-0.11274
RTN3	miR-484	-0.10879
BCLAF1	miR-423-5p	-0.10727
WRAP53	miR-455-3p	-0.10259
CHCHD3	miR-423-3p	-0.09823
EXOC7	miR-331-3p	-0.09759
SALL2	miR-484	-0.08909
GNB2	miR-331-3p	-0.08692
LRCH4	miR-423-5p	-0.08192
EDC3	miR-324-5p	-0.07175
MED22	miR-331-3p	-0.06909
CRB2	miR-342-3p	-0.06854
UBE2Q2	miR-331-3p	-0.06283
TSPAN3	miR-484	-0.05973
BOP1	miR-324-5p	-0.05796
TMED9	miR-484	-0.05366
BAIAP2	miR-455-3p	-0.04032
MACF1	miR-125a-5p	-0.03858

COL6A1	miR-324-5p	-0.03834
GNAS	miR-324-3p	-0.03427
SF3B2	miR-331-3p	-0.03314
FXR2	miR-324-3p	-0.03189
NPTX2	miR-423-3p	-0.02829
SMG6	miR-331-3p	-0.02545
RPN2	miR-484	-0.01973
MICALL1	miR-423-5p	-0.01794
KREMEN1	miR-484	-0.01387
ZFC3H1	miR-342-3p	-0.01109
TJAP1	miR-331-3p	-0.00832
MED14	miR-532-3p	-0.00660
RFT1	miR-330-5p	-0.00233
DDX11	miR-324-5p	-0.00214
TUBGCP6	miR-324-5p	0.00373
REXO1	miR-484	0.01123
ARMC6	miR-484	0.01136
TBC1D2B	miR-484	0.01242
CBX1	miR-342-3p	0.01251
GRK6	miR-361-5p	0.02378
SS18	miR-331-3p	0.02542
PGD	miR-339-5p	0.02912
EEF1A1	miR-361-3p	0.02978
CPSF2	miR-193a-3p	0.03072
PFKL	miR-423-3p	0.03324
CLSTN1	miR-423-5p	0.03771
CCDC47	miR-484	0.03959
RANGAP1	miR-331-3p	0.04404

ZBTB34	miR-423-5p	0.04460
PWP2	miR-342-3p	0.04651
KPNB1	miR-455-3p	0.04927
TMEM9B	miR-484	0.05482
TNRC6B	miR-484	0.05515
BLMH	miR-484	0.05830
TXLNA	miR-484	0.06246
XRCC6	miR-484	0.06297
EMD	miR-484	0.06487
AKAP13	miR-423-5p	0.06657
RASL10B	miR-331-3p	0.06777
SRF	miR-331-3p	0.06979
RANBP10	miR-330-5p	0.07211
ARAF	miR-331-3p	0.07236
TFCP2	miR-331-3p	0.07380
TARS2	miR-484	0.07728
GOT2	miR-484	0.08313
GJA1	miR-342-3p	0.08391
MSL3	miR-484	0.08508
PIN1	miR-484	0.08634
CYB5R3	miR-484	0.08708
BAHD1	miR-324-3p	0.09225
NGRN	miR-342-3p	0.09600
KLHL15	miR-532-3p	0.09640
CASP7	miR-484	0.09814
POLR2L	miR-423-3p	0.09881
NUBP1	miR-423-3p	0.09891
CCND2	miR-342-3p	0.09913

TIA1	miR-331-3p	0.09921
GLDC	miR-423-3p	0.10023
HMGA1	miR-331-3p	0.10341
TUBGCP4	miR-484	0.11117
FANCD2	miR-331-3p	0.12052
PLOD1	miR-484	0.12160
SCAP	miR-484	0.12556
PARM1	miR-324-5p	0.13679
DHCR24	miR-484	0.13725
HSP90B1	miR-484	0.13814
POLD1	miR-324-5p	0.13830
FLT4	miR-331-3p	0.13862
EXOG	miR-484	0.14110
SREBF1	miR-484	0.14223
ANXA5	miR-330-5p	0.14269
RRM2	miR-484	0.14340
NANS	miR-324-5p	0.15372
USP10	miR-455-3p	0.15378
MID2	miR-324-5p	0.15425
EZH2	miR-484	0.15832
MPP2	miR-484	0.16015
PML	miR-423-3p	0.16110
TBCB	miR-324-5p	0.16501
NUCB1	miR-484	0.16620
SLC4A2	miR-455-3p	0.16770
CYCS	miR-361-5p	0.16816
HSPBP1	miR-484	0.16933
TRERF1	miR-324-5p	0.17515

PUM1	miR-324-3p	0.17730
BACE2	miR-484	0.18217
CIAPIN1	miR-361-3p	0.18732
UBTF	miR-484	0.18919
HNRNPM	miR-331-3p	0.19242
GHITM	miR-484	0.19261
PSMD7	miR-331-3p	0.19707
TIPIN	miR-484	0.20188
PLA2G4F	miR-125a-5p	0.20339
GAPDH	miR-324-3p	0.21235
POLDIP3	miR-331-3p	0.21673
FOSL2	miR-342-3p	0.21786
FASN	miR-484	0.21832
FLII	miR-331-3p	0.22278
SLC11A2	miR-484	0.22402
PSMD6	miR-331-3p	0.22674
KDM2B	miR-324-3p	0.22716
DYRK2	miR-484	0.22780
TNFRSF12A	miR-484	0.22790
POLR3K	miR-484	0.22864
SCMH1	miR-532-3p	0.22986
USP5	miR-455-3p	0.23068
GNB2	miR-324-5p	0.23170
FIGN	miR-342-3p	0.23178
AGAP1	miR-324-5p	0.23410
PTP4A2	miR-331-3p	0.24057
INTS4	miR-455-3p	0.24118
CCDC97	miR-484	0.24176

UBE2I	miR-324-5p	0.24287
PTK7	miR-324-5p	0.24413
FAM3C	miR-342-3p	0.24720
UBE4B	miR-423-3p	0.25010
SMC1A	miR-339-5p	0.25102
APRT	miR-324-3p	0.25394
ATP2A2	miR-484	0.25743
LAMB1	miR-125a-5p	0.25911
CHTF18	miR-484	0.25973
PSMD1	miR-423-3p	0.26014
ACLY	miR-484	0.26526
CLN6	miR-324-5p	0.26849
PMS2	miR-324-5p	0.27077
ANKRD13D	miR-331-3p	0.27264
DRAP1	miR-423-5p	0.27440
PFAS	miR-324-5p	0.27566
FDX1	miR-455-3p	0.27610
TDRKH	miR-423-5p	0.27646
FADS2	miR-423-3p	0.28407
RRBP1	miR-331-3p	0.28785
NCLN	miR-331-3p	0.28923
HNRNPH2	miR-484	0.29265
GBA2	miR-455-3p	0.29444
ACTG1	miR-342-3p	0.29580
RABAC1	miR-423-5p	0.30147
YWHAZ	miR-484	0.30883
SMO	miR-331-3p	0.31002
SH3BP2	miR-331-3p	0.31614

HNRNPA1	miR-339-5p	0.31752
LDHB	miR-324-5p	0.31946
STMN1	miR-484	0.32381
KCTD2	miR-423-5p	0.32427
DDX54	miR-484	0.32561
DLG5	miR-484	0.32778
ATXN7L3B	miR-484	0.33243
GATA6	miR-140-5p	0.33375
CHD8	miR-423-5p	0.33384
EXOC6B	miR-331-3p	0.33679
MDH2	miR-484	0.34253
HGS	miR-423-3p	0.34276
ANAPC1	miR-484	0.34319
XPOT	miR-342-3p	0.34493
SLC10A7	miR-484	0.34881
GNPAT	miR-331-3p	0.34964
USF2	miR-339-5p	0.35238
CNOT2	miR-361-5p	0.35249
SLC9A3R1	miR-423-5p	0.35316
CRY2	miR-532-3p	0.35483
APP	miR-423-3p	0.35513
WBP2	miR-324-5p	0.35592
ANXA6	miR-324-5p	0.35835
CAD	miR-455-3p	0.36287
MTHFD1	miR-324-5p	0.36514
MAP4	miR-331-3p	0.36765
NDE1	miR-423-3p	0.36782
GOT2	miR-342-3p	0.36786

MIER2	miR-423-5p	0.37112
PRKCE	miR-342-3p	0.37582
ATP6V1E1	miR-423-5p	0.37901
HMGXB3	miR-324-5p	0.37908
VPS18	miR-324-3p	0.38240
CXXC1	miR-324-3p	0.38301
PSMD2	miR-331-3p	0.38547
SF3B3	miR-324-3p	0.39445
MED15	miR-324-3p	0.39448
BOK	miR-331-3p	0.39528
MLLT1	miR-484	0.39759
SLC4A2	miR-484	0.39886
GALM	miR-455-3p	0.40060
SPEN	miR-324-5p	0.40065
CRTAP	miR-484	0.40175
DNMT1	miR-484	0.40199
UCK2	miR-361-5p	0.40322
KDM5C	miR-484	0.40332
TRERF1	miR-342-3p	0.40453
EXOSC10	miR-484	0.40568
NHP2	miR-330-5p	0.40932
PXDN	miR-324-5p	0.41359
HIPK1	miR-484	0.41773
ATP2B4	miR-423-5p	0.41873
JAG2	miR-484	0.41979
TCOF1	miR-324-5p	0.42182
DDX23	miR-361-5p	0.42339
HYOU1	miR-484	0.42401

IRAK1	miR-423-3p	0.42492
PHGDH	miR-331-3p	0.42535
LDHA	miR-331-3p	0.42587
PSMD2	miR-361-5p	0.43131
FASN	miR-331-3p	0.43436
KIF1A	miR-423-5p	0.43448
FLII	miR-423-3p	0.43489
CDS2	miR-484	0.43827
FBXL5	miR-331-3p	0.43829
TFE3	miR-455-3p	0.43875
SF3B4	miR-423-3p	0.43924
FRMD4A	miR-423-3p	0.44483
TBC1D22A	miR-484	0.44631
SP3	miR-484	0.44818
DGKZ	miR-331-3p	0.44834
UCP2	miR-484	0.44929
CEBPA	miR-193a-5p	0.44962
ASXL1	miR-484	0.45026
MED18	miR-532-3p	0.45101
FLNA	miR-331-3p	0.45133
ACTA2	miR-484	0.45481
MAPRE2	miR-455-3p	0.45637
PIAS4	miR-324-5p	0.45864
MRPL3	miR-484	0.45957
HPS1	miR-331-3p	0.46207
NPEPPS	miR-484	0.46299
TNKS	miR-324-5p	0.46447
USP5	miR-484	0.46969

TOR1AIP2	miR-455-3p	0.47414
XRCC5	miR-331-3p	0.47436
RAB11FIP1	miR-324-5p	0.47681
RCE1	miR-484	0.47857
EIF4G2	miR-331-3p	0.47896
PSMD1	miR-331-3p	0.47955
ZER1	miR-331-3p	0.48262
ABCC5	miR-423-5p	0.48312
PTPRF	miR-455-3p	0.48569
DHX40	miR-484	0.48677
DGCR2	miR-484	0.48750
HNRNPM	miR-484	0.48879
RUVBL2	miR-324-3p	0.49071
INTS5	miR-484	0.49096
ACTB	miR-324-5p	0.50154
IQGAP1	miR-324-3p	0.50348
MTR	miR-484	0.50688
IDS	miR-342-3p	0.50898
FAM160B2	miR-484	0.50959
MTHFD1L	miR-484	0.51221
SNX7	miR-324-5p	0.51355
HNRNPF	miR-339-5p	0.51529
SLC25A14	miR-331-3p	0.51699
CHPF2	miR-339-5p	0.52170
PI4KB	miR-331-3p	0.53425
TUBA1C	miR-324-5p	0.53538
EPN1	miR-423-5p	0.53624
ZBTB4	miR-331-3p	0.53665

ACLY	miR-361-5p	0.53765
COL14A1	miR-324-5p	0.54237
LRRC20	miR-484	0.54278
CSTB	miR-423-5p	0.54419
XRCC6	miR-324-3p	0.55109
SEMA3D	miR-193a-3p	0.55141
PHKA1	miR-484	0.55277
HYOU1	miR-455-3p	0.55436
FTSJ3	miR-330-5p	0.55457
OSTC	miR-324-5p	0.55877
LRRC8B	miR-423-3p	0.55955
SLC30A5	miR-361-5p	0.56118
GAPDH	miR-484	0.56453
TEP1	miR-484	0.56871
ACIN1	miR-324-5p	0.56888
ARF1	miR-324-5p	0.57000
AXIN1	miR-484	0.57278
EEF1A1	miR-339-5p	0.57362
TMUB1	miR-423-5p	0.57633
CHAF1B	miR-484	0.57709
AKT3	miR-146b-5p	0.57895
TMEM8B	miR-324-5p	0.58017
RRAGD	miR-484	0.58198
SYK	miR-331-3p	0.58293
CFL1	miR-324-5p	0.58755
CDCA7L	miR-484	0.59133
PPP2R5E	miR-125a-5p	0.59156
KLHDC3	miR-324-5p	0.59860

AKAP13	miR-484	0.60017
ATXN7L3	miR-125a-5p	0.60030
GIGYF2	miR-455-3p	0.60390
NOLC1	miR-423-5p	0.60566
GTF3C1	miR-484	0.60578
CFL1	miR-484	0.60725
AKIRIN1	miR-331-3p	0.61067
SNX11	miR-484	0.61068
CYFIP2	miR-423-3p	0.61682
PTPN13	miR-331-3p	0.62076
SPTBN1	miR-423-3p	0.62519
XRCC6	miR-423-3p	0.63185
CD2AP	miR-484	0.63861
SRRM2	miR-455-3p	0.64132
SON	miR-455-3p	0.64269
GTF2H3	miR-484	0.64721
CTNNB1	miR-331-3p	0.64949
MAP4	miR-484	0.65377
CLEC16A	miR-331-3p	0.65468
VPS4A	miR-455-3p	0.65728
FAM117A	miR-484	0.65766
SLC39A8	miR-331-3p	0.65969
SPIRE1	miR-484	0.66350
SON	miR-361-5p	0.66513
RAP2B	miR-324-5p	0.66516
TCF19	miR-484	0.66968
TROAP	miR-484	0.67836
WNK1	miR-331-3p	0.67867

NOTCH3	miR-484	0.67867
TUT1	miR-193a-3p	0.68800
CHSY3	miR-484	0.68921
BRCA1	miR-484	0.69097
TUBA1B	miR-324-5p	0.69443
RUNX2	miR-484	0.69977
ACTR1A	miR-331-3p	0.70208
PPARD	miR-331-3p	0.70508
DRG1	miR-455-5p	0.70785
MSH6	miR-324-5p	0.70816
PGAM1	miR-324-5p	0.70821
RPS5	miR-455-3p	0.71093
UBE2Z	miR-484	0.71350
SREBF2	miR-331-3p	0.71752
SCML2	miR-423-3p	0.71771
CEP78	miR-423-3p	0.72251
MED15	miR-484	0.72409
FLOT1	miR-484	0.72535
UBA1	miR-331-3p	0.72598
RPL8	miR-455-3p	0.72733
PEPD	miR-423-3p	0.73390
KNTC1	miR-484	0.73507
BAD	miR-330-5p	0.74148
TRIM28	miR-423-5p	0.74587
BTBD2	miR-324-5p	0.74716
ARHGAP17	miR-324-3p	0.74811
KLC2	miR-331-3p	0.74876
MAP2K7	miR-125a-5p	0.74909

CWC22	miR-484	0.75275
MYO9B	miR-484	0.75342
ASS1	miR-324-5p	0.75721
MYO5A	miR-484	0.75809
FURIN	miR-484	0.75890
AGK	miR-484	0.76318
FYCO1	miR-484	0.76869
RPS2	miR-455-3p	0.77309
EEF1A1	miR-125a-5p	0.77605
TBRG1	miR-484	0.77621
UBQLN2	miR-484	0.78200
BCL2L12	miR-455-3p	0.78288
N4BP2L2	miR-455-3p	0.79396
TUFT1	miR-330-5p	0.79505
PLXNB2	miR-331-3p	0.80178
PREPL	miR-125a-5p	0.80259
CBS	miR-324-5p	0.80374
RFX3	miR-342-3p	0.80880
HCLS1	miR-484	0.81417
AP2A2	miR-331-3p	0.81497
KIF3B	miR-324-5p	0.81886
ITM2C	miR-324-5p	0.81960
TBC1D10B	miR-324-5p	0.82071
CLCN7	miR-125a-5p	0.82246
MCM4	miR-324-3p	0.82345
PFKL	miR-484	0.82378
WBP2	miR-361-5p	0.82476
FAM168B	miR-331-3p	0.82649

CDS1	miR-324-5p	0.82827
PPM1F	miR-324-5p	0.83477
PGAM1	miR-484	0.83596
MSI2	miR-484	0.83625
NFE2L1	miR-484	0.84118
FGFBP3	miR-484	0.84154
IRS2	miR-484	0.84374
BBX	miR-484	0.84374
ENO1	miR-484	0.84470
DDX18	miR-324-3p	0.84984
TRAFD1	miR-324-5p	0.85198
HNRNPC	miR-484	0.85318
RNF26	miR-324-5p	0.85757
TMEM115	miR-455-3p	0.85779
HNRNPA1	miR-324-5p	0.85801
ORAI2	miR-484	0.85868
DYRK1B	miR-324-5p	0.85949
HK2	miR-484	0.85979
ZDHHC9	miR-125a-5p	0.86848
RPS15A	miR-455-3p	0.87341
RFX5	miR-484	0.88081
AP1B1	miR-484	0.88723
ATN1	miR-423-5p	0.88796
ZFX	miR-484	0.88824
NEO1	miR-484	0.89283
MLH3	miR-484	0.89346
UBA1	miR-324-5p	0.89369
THRAP3	miR-125a-5p	0.89648

AMMECR1L	miR-484	0.90135
PLD3	miR-484	0.90738
KLC2	miR-484	0.90892
MAPKAPK2	miR-324-5p	0.91362
PHLPP1	miR-484	0.91483
ADAM10	miR-484	0.92008
ITGA9	miR-484	0.92283
SNRPB2	miR-193a-3p	0.92378
PRKCD	miR-484	0.92477
SEMA4D	miR-484	0.92866
AKT1S1	miR-484	0.92875
ARNT	miR-324-5p	0.93832
PLS3	miR-125a-5p	0.95874
CAPRIN1	miR-484	0.96027
FKBP4	miR-423-5p	0.97889
